# Genomic adaptations to cereal‐based diets contribute to mitigate metabolic risk in some human populations of East Asian ancestry

**DOI:** 10.1111/eva.13090

**Published:** 2020-09-08

**Authors:** Arianna Landini, Shaobo Yu, Guido Alberto Gnecchi‐Ruscone, Paolo Abondio, Claudia Ojeda‐Granados, Stefania Sarno, Sara De Fanti, Davide Gentilini, Anna Maria Di Blasio, Hanjun Jin, Thanh Tin Nguyen, Giovanni Romeo, Cecilia Prata, Eugenio Bortolini, Donata Luiselli, Davide Pettener, Marco Sazzini

**Affiliations:** ^1^ Laboratory of Molecular Anthropology & Centre for Genome Biology Department of Biological, Geological and Environmental Sciences University of Bologna Bologna Italy; ^2^ Centre for Global Health Research Usher Institute of Population Health Sciences and Informatics University of Edinburgh Edinburgh UK; ^3^ Department of Archaeogenetics Max Planck Institute for the Science of Human History Jena Germany; ^4^ Department of Molecular Biology in Medicine Civil Hospital of Guadalajara “Fray Antonio Alcalde” and Health Sciences Center University of Guadalajara Guadalajara Mexico; ^5^ Interdepartmental Centre Alma Mater Research Institute on Global Challenges and Climate Change University of Bologna Bologna Italy; ^6^ Department of Brain and Behavioral Sciences University of Pavia Pavia Italy; ^7^ Italian Auxologic Institute IRCCS Cusano Milanino, Milan Italy; ^8^ Department of Biological Sciences College of Natural Science Dankook University Cheonan South Korea; ^9^ Hue University of Medicine and Pharmacy Hue Vietnam; ^10^ Medical Genetics Unit S. Orsola Hospital University of Bologna Bologna Italy; ^11^ European School of Genetic Medicine Italy; ^12^ Department of Pharmacy and Biotechnology University of Bologna Bologna Italy; ^13^ Department of Cultural Heritage University of Bologna Ravenna Italy

**Keywords:** dietary selective pressures, evolutionary medicine, genomic adaptation, human Asian populations, metabolic risk

## Abstract

Adoption of diets based on some cereals, especially on rice, signified an iconic change in nutritional habits for many Asian populations and a relevant challenge for their capability to maintain glucose homeostasis. Indeed, rice shows the highest carbohydrates content and glycemic index among the domesticated cereals and its usual ingestion represents a potential risk factor for developing insulin resistance and related metabolic diseases. Nevertheless, type 2 diabetes and obesity epidemiological patterns differ among Asian populations that rely on rice as a staple food, with higher diabetes prevalence and increased levels of central adiposity observed in people of South Asian ancestry rather than in East Asians. This may be at least partly due to the fact that populations from East Asian regions where wild rice or other cereals such as millet have been already consumed before their cultivation and/or were early domesticated have relied on these nutritional resources for a period long enough to have possibly evolved biological adaptations that counteract their detrimental side effects. To test such a hypothesis, we compared adaptive evolution of these populations with that of control groups from regions where the adoption of cereal‐based diets occurred many thousand years later and which were identified from a genome‐wide dataset including 2,379 individuals from 124 East Asian and South Asian populations. This revealed selective sweeps and polygenic adaptive mechanisms affecting functional pathways involved in fatty acids metabolism, cholesterol/triglycerides biosynthesis from carbohydrates, regulation of glucose homeostasis, and production of retinoic acid in Chinese Han and Tujia ethnic groups, as well as in people of Korean and Japanese ancestry. Accordingly, long‐standing rice‐ and/or millet‐based diets have possibly contributed to trigger the evolution of such biological adaptations, which might represent one of the factors that play a role in mitigating the metabolic risk of these East Asian populations.

## 1 INTRODUCTION

During the recent evolutionary history of *H. sapiens*, several dietary shifts are supposed to have introduced new selective pressures on human groups from different ecoregions, especially due to domestication of a variety of plants and animals (Bayoumi et al., 2016; Brown, 2012; Luca, Perry, & Di Rienzo, 2010; Lucock, 2007; Sazzini et al., 2016, 2020). For instance, the adoption of a diet based on cereals, especially on rice, represented an iconic change in nutritional habits for many Asian populations and one of the most relevant challenges experienced by the human metabolism. In fact, this determined a remarkable increase in the intake of carbohydrate‐rich foods and a shift toward a high glycemic load (GL) diet, which is expected to have affected the capability of these populations to maintain a physiological glucose homeostasis. To this end, unpolished brown rice can be considered as a reliable proxy for the rice varieties long consumed by the ancestors of Asian people and both its traditional and modern cultivars present some of the highest carbohydrates content and glycemic index (GI) among the domesticated cereals (Fitzgerald et al., 2011). Indeed, by considering different varieties it shows on average GI = 88 ± 5 and GL = 20, while for example wheat GI and GL are attested respectively to 30 ± 9 and 11 (Atkinson, Foster‐Powell, & Brand‐Miller, 2008). This GI/GL difference between rice and other grains is even more striking when considering the polished white rice (GI = 103 ± 11, GL = 29), which is consumed as a staple food by modern Asian populations. Therefore, rice has long acted and is still acting as the primary contributor to the dietary GL of Asian groups. By prompting rapid postprandial increase in glycemia, its usual and massive ingestion represents one of the potential risk factors for developing insulin resistance and related metabolic diseases, such as type 2 diabetes (T2D) and obesity (Boers, Seijen Ten Hoorn, & Mela, 2015; Hu, Pan, Malik, & Sun, 2012).

This is consistent with the fact that after the changes in dietary habits and lifestyle experienced by most Asian populations in the last decades, the number of individuals affected by T2D in these human groups has rapidly increased, until representing around 60% of the world's diabetic subjects (Hu, 2011). The fast socio‐economic transitions that have interested Asian countries have indeed led to a further increment in the intake of calories and high‐GI foods due to augmented consumption of oils, fats, refined grains and sugars, dairy and convenience foods (Kelly, 2016). This was coupled also with the decrease in physical activity due to urbanization, adoption of sedentary jobs, and motorized transports (Ramachandran, Chamukuttan, Shetty, Arun, & Susairaj, 2012; Ramachandran & Snehalatha, 2010; Ramachandran et al., 2004; Wu, 2006; Yoon et al., 2006). In particular, substantial shifts from traditional to even more caloric and high‐GL westernized‐type dietary patterns are expected to have exacerbated metabolic risk in East Asian groups such as those from China and Japan. Indeed, these populations considerably increased their utilization of dairy, butter, and meat, along with the consumption of high‐GI foods, such as confectionaries, jam, fruit, sugars, bread, noodles, and potatoes (Gabriel, Ninomiya, & Uneyama, 2018; Murakami et al., 2006; Popkin, Horton, Kim, Mahal, & Shuigao, 2001; Tomisaka et al., 2002). For instance, Chinese and Japanese populations present a daily energy intake ranging from around 2,400 to 2,800 kcal. This is largely ascribable to an average consumption of respectively 450 g and 286 g of cereals per day (~62% of which represented by rice) and to a remarkable increase in the proportion of dietary fats (on average 26.4% of the total calorie entry). Moreover, as many other East Asian groups they have recently gone through an escalation of the per capita consumption of sugars (on average ~50 g/day) (Murakami et al., 2006; Food and Agriculture Organization of the United Nations, 2008; Zhai et al., 2009; Fujiwara et al., 2018). A similar, but quantitatively less pronounced nutrition transition has been experienced also by populations from the Indian subcontinent. In fact, they show lower daily energy intake (2,020–2,047 kcal), cereals consumption (368 g per day, ~52% of which represented by rice), and percentage of dietary fats (16%–21%) than Chinese and Japanese groups (Dixit, Azar, Gardner, & Palaniappan, 2011; Misra et al., 2011; Radhika, Van Dam, Sudha, Ganesan, & Mohan, 2009; Bharati and Kulkarni, 2020). Conversely, they consume more fiber (13.3 g/day) with respect to Chinese and Japanese people (9.7 g/day and 7.6 g/day, respectively) and a slightly higher amount of sugars (55.3 g/day) (Dasgupta, Pillai, Kumar, & Arora, 2015). Nevertheless, per capita sugar consumption was found to be significantly correlated with T2D prevalence in Central and South East Asian populations, but not in South Asian and East Asian ones, suggesting that other factors may contribute mostly to the high‐GL diet of these human groups (Praveen, Sayumi, Yashasvi, Ganga, & Saroj, 2014).

Interestingly, in contrast to what expected according to the described dietary shifts, increasing rates in the prevalence of T2D were found to be substantially higher in South Asian populations rather than in East Asians (e.g., 12.1% and 10.6% in India and Pakistan versus 6.1% and 7.6% in China and Korea) (Chan et al., 2009; Ma & Chan, 2013; Ramachandran, Ma, & Snehalatha, 2010; Yoon et al., 2006). These peculiar epidemiological patterns may suggest that when coupled with recent transitions in lifestyle and dietary habits, and with subsequent increases in life expectancy, cereal‐based diets, particularly those relying on rice as a staple food, might have further increased T2D/obesity susceptibility especially in populations from the Indian subcontinent. In fact, it seems that an analogous increase in metabolic risk is somehow mitigated at least in some East Asian groups, suggesting that their ancestors might have evolved adaptations triggered by several millennia of abundant rice (or other cereals) consumption that contribute to reduce the potential side effects of medium‐to‐high GI foods.

In particular, we propose that populations from East Asian regions where wild rice (*Oryza rufipogon*) originated and was early domesticated have heavily relied on such a nutritional resource for a sufficient time (i.e., around 10,000 years) to have evolved genetic adaptations to it. In this regard, the Yangtze River valley in Eastern China is the geographical area where the oldest archaeological evidence of habitual wild rice consumption (dated to 12,000–11,000 years ago) was found (Jiang & Liu, 2006; Zhao, 1998) and where the modern rice subspecies *O. sativa japonica* was domesticated (Gross & Zhao, 2014; Silva et al., 2015, 2018). Accordingly, human groups from this region are supposed to have introduced rice in their diet long before its domestication and the development of cultivation techniques, which have been established 7,000–6,000 years ago after an extended period of predomesticated cultivation and a much longer period of wild rice use by foragers (Cao et al., 2006; Fuller, Harvey, & Qin, 2007). Afterward, rice agriculture diffused from China to the Korean peninsula and Japan, replacing the indigenous cultivations of millet and beans (Gross & Zhao, 2014). A similar pattern might be hypothesized also for broomcorn millet (*Panicum miliaceum*) and foxtail millet (*Setaria italica*), whose domestication was completed 8,000–6,000 years ago in the Hebei and Manchuria provinces of Northern China (Deng, Hung, Fan, Huang, & Lu, 2018; Stevens et al., 2016; Zhao, 2011), and whose cultivation was in parallel established in nearby Korean and Japanese regions (Gross & Zhao, 2014). Conversely, wheat consumption has played a marginal role in the diet of ancestral East Asian populations, having been introduced only 4,000 years ago and having remained circumscribed to few Chinese regions at least up to the 6th century AD (Zhou & Garvie‐Lok, 2015). Moreover, it long represented a prerogative of a small fraction of these populations given that wheat was generally not cultivated as a high production subsistence but was instead consumed as a rare exotic good by social elites (Liu & Jones, 2014; Long et al., 2018).

An independent center of rice domestication but involving a different subspecies (i.e., *O. sativa indica*) has been identified also in the Indo‐Gangetic Plain crossing northern regions of the Indian subcontinent (Molina et al., 2011). However, such a domestication process was completed only several thousand years later with respect to what occurred in the Yangtze River Valley, when the subspecies *O. sativa japonica* was introduced from China and hybridized with the local proto‐*indica* rice (Choi et al., 2017; Gross & Zhao, 2014; Silva et al., 2018). Furthermore, *O. sativa japonica* was selected by East Asian farmers in response to specific dietary habits and this resulted in its stickier grains and glutinous phenotype, which are associated also to low amylose levels (0%–20%) (Olsen et al., 2006; Yamanaka, Nakamura, Watanabe, & Sato, 2004). Conversely, the preference for distinct, noncohesive rice grains in South Asian cultures prevented the introgression of these traits into the *indica* variety (Fuller and Castillo, 2016), which is indeed characterized by a higher amount of amylose (23%–31%) than *O. sativa japonica* (Kaur, Ranawana, & Henry, 2016). Interestingly, inverse correlation between rice amylose content and GI was observed, with East Asian cultivars showing remarkably higher GI (on average ~ 100) with respect to South Asian ones (average GI = 60) (Fitzgerald et al., 2011), thus having potentially represented a more challenging selective pressure for the human metabolism.

Overall, these evidences suggest that the ancestors of modern Chinese, Korean, and Japanese populations have adopted high‐GL cereal‐based diets for a period of time considerably longer than other East Asian and South Asian human groups. Therefore, they might have had the opportunity to evolve genetic adaptations in response to such a peculiar dietary regimen. To test such a hypothesis, we assembled a genome‐wide “Pan‐Asian” dataset including 2,379 individuals from 124 East Asian and South Asian populations selected to be representative of human genetic variation observable at geographical areas where wild rice or millet originated and/or were early domesticated (i.e., candidate populations) or where the adoption of cereal‐based diets occurred only several thousand years later (i.e., control populations) (Table S1). We then identified genetically homogenous population clusters, and we investigated their adaptive evolution by searching for genomic signatures ascribable to the action of natural selection according to models of hard/soft selective sweeps (i.e., events of strong natural selection on single/few loci with large effects on a given phenotypic trait) and polygenic adaptation (i.e., events of weak, but pervasive natural selection on many loci functionally related with each other, but individually showing small effects on a given phenotypic trait). This enabled us to shortlist adaptive events at several genes playing a role primarily in fatty acids metabolism, regulation of glucose homeostasis and production of retinoic acid, which have been possibly evolved in response to rice‐ and/or millet‐based diets by Chinese populations of Han and Tujia ancestry, Korean and Japanese people. Therefore, we provided evidence for genetic adaptations of these human groups that might contribute to mitigate the side effects of their diets, thus shedding new light on the possible evolutionary causes having partly influenced the present‐day epidemiological patterns of metabolic diseases observed across the Asian continent.

## 2 METHODS

### 2.1 Samples collection and genotyping

Saliva from 85 individuals representative of Korean (*N* = 36), Bangladeshi (*N* = 29), and Vietnamese (*N* = 20) populations was sampled using the Oragene DNA OG‐500 kit (DNA Genotek, Ottawa, Ontario, Canada) and according to ethnographic information. Informed consent was also obtained from all individuals, and on 04/08/2013 the University of Bologna ethics committee released approval for the present study (within the framework of the ERC‐2011‐AdG 295733 project), which was designed and conducted in accordance with relevant guidelines and regulations and according to ethical principles for medical research involving human subjects stated by the WMA Declaration of Helsinki. DNA was extracted from saliva samples using the *prepit‐L2P* protocol (DNA Genotek, Ottawa, Ontario, Canada) and quantified with the Quant‐iT dsDNA Broad‐Range Assay Kit (Invitrogen Life Technologies, Carlsbad, CA, USA). DNA samples were genotyped for ~720,000 genome‐wide SNPs by means of the HumanOmniExpress v 1.1 chip (Illumina, San Diego, CA, USA) at the Center for Biomedical Research & Technologies of the Italian Auxologic Institute (Milan, Italy).

### 2.2 Data curation and assembly of a Pan‐Asian dataset

Quality control (QC) procedures were performed on the generated genome‐wide data using PLINK v.1.9 (Purcell et al., 2007). Only autosomal loci with a genotyping success rate higher than 95% and no significant deviations from the Hardy–Weinberg equilibrium (*p* > 1.41 × 10^−8^) were retained. Individuals showing more than 5% of missing data were excluded, together with ambiguous strand single nucleotide polymorphisms (SNPs) with A/T or G/C alleles. The obtained dataset was then submitted to linkage‐disequilibrium (LD) pruning, and the identified SNPs in approximate LD with each other (*r* ^2^ < .1) were used to estimate the degree of recent shared ancestry (IBD) for each pair of subjects by calculating the genome‐wide proportion of shared alleles (i.e., identity by state, IBS). Individuals showing IBD kinship coefficient higher than 0.1 were removed. A “high‐quality” dataset of 82 samples typed for 688,987 SNPs was thus generated. An “extended” dataset of 4,356 samples belonging to 162 worldwide populations typed for 231,947 SNPs was also obtained by merging the “high‐quality” dataset with publicly available data retrieved from the 1,000 Genomes Project phase 3 (1000 Genomes Project Consortium et al., 2015), the HGDP project (Li et al., 2008), as well as literature studies focused on South Asian and East Asian groups (Table S1). Such an “extended” dataset was prepared for population structure and admixture analyses by LD‐pruning (*r* ^2^ < .2) and by removing sites with a minor allele frequency (MAF) below 0.01. Haplotypes phasing was performed with SHAPEIT2 v2.r790 (Delaneau, Coulonges, & Zagury, 2008) using default parameters and HapMap phase 3 recombination maps.

### 2.3 Population structure analyses

Principal component analysis (PCA) was applied first to the “extended” dataset to check for the presence of potential genotypes inconsistency due to errors occurred in the merging procedure and then to a “Pan‐Asian” subset of 2,379 individuals belonging to 124 Asian populations. To compute PCA, the *smartpca* method implemented in the EIGENSOFT package v6.0.1 (Patterson, Price, & Reich, 2006) was used. To depict an overall picture of ancestry proportions for each subject belonging to a further representative subset of 1,171 samples from 57 Asian populations selected according to PCA results, and to test for the presence of genetically homogeneous population clusters, we applied the model‐based clustering algorithm implemented in ADMIXTURE v1.22 (Alexander, Novembre, & Lange, 2009) assuming *K* = 2 to *K* = 10 clusters. This infers the relative proportions of the different genetic components observable within each genome and which are due to the gene flow that generally occurred between human groups. Therefore, although being formally a clustering analysis, it is commonly used to explore patterns of shared genetic ancestry among populations. We ran 50 replicates with different random seeds for each K to monitor for convergence and only those presenting the highest log‐likelihood values were plotted. Concurrently, we calculated cross‐validation (CV) errors for each K to identify the most plausible number of ancestry components.

### 2.4 Selection scans on the identified population clusters

We computed the number of segregating sites by length (nSL) statistics (Ferrer‐Admetlla, Liang, Korneliussen, & Nielsen, 2014) to investigate both hard and soft selective sweeps occurred in the genomes ascribable to the clusters pointed out by population structure analyses. The nSL test quantifies the extension of haplotype homozygosity around each of the considered SNPs and compares the patterns obtained for haplotypes carrying the ancestral allele or the derived allele to pinpoint genomic regions that have been plausibly subjected to natural selection. Information about the ancestral and derived alleles at each SNPs was retrieved by considering the human ancestor alignment, which was obtained through multiple alignment of six primate genomes to the human reference sequence build 37 (UCSC hg19). We then used algorithms implemented in Selscan v1.1.0b (Szpiech & Hernandez, 2014) to compute nSL scores for each SNP by considering a 20,000 bp threshold for gap scale and 200,000 bp as the maximum length a gap can reach, otherwise the calculation was aborted. Since the nSL statistics measures the distance between SNPs in terms of number of polymorphic sites across the considered genomic region, we set a maximum extension of 25 SNPs according to the SNPs density of our dataset. Unstandardized nSL scores were normalized by classifying SNPs in frequency bins across the genome and by subtracting to each value the mean nSL score in that bin and by dividing by the associated standard deviation.

### 2.5 Shortlisting of the most informative candidate adaptive genes

To minimize false‐positive results (i.e., isolated SNPs showing unusual nSL scores) that might be ascribable to stochastic variation in allele frequency due to genetic drift, we applied a 200,000 bp sliding windows procedure on the obtained genome‐wide nSL distribution. Derived allele frequency (DAF) of each SNP was computed to enable their classification into bins based on DAF (Piras et al., 2012). We retained outlier SNPs in a given genomic window as those showing absolute nSL values falling within the 99th percentile of the related DAF‐based bin distribution, and we further classified windows into bins according to their total number of SNPs. Consecutive windows were merged together, and the top 1% of windows for each bin of regions was extracted according to their proportion of outlier SNPs with respect to the overall number of SNPs in such a window. This enabled us to detect large genomic segments enriched for unusual nSL scores. The HumanOmniExpress bead chip annotation file (Illumina, San Diego, CA, USA) was used to assign genomic regions enriched for outlier SNPs to known genes. For each population clusters, we then investigated known functional relationships between genes included in the top 1% windows by using information from the STRING v10.5 protein–protein interactions database (Jensen et al., 2009). By assigning to each SNP the ranking value previously calculated for the respective window, a final ranking of merged chromosomal intervals was obtained according to the computation of the arithmetical mean of scores of the windows involved. Finally, the most informative candidate adaptive genes for each population cluster were identified as those included in the top 1% merged windows showing a proportion of outlier SNPs above 0.5.

### 2.6 Gene network analyses aimed at testing for polygenic adaptation

Population clusters pointed out by the performed selection scan as the most plausible groups having evolved adaptations in response to rice or millet‐based diets were further investigated to explicitly test the occurrence of selective events according to a polygenic adaptation model. This assumes that adaptive traits have been modulated by weak positive selection acting concurrently on multiple loci belonging to specific gene networks rather than on single genes (Gnecchi‐Ruscone et al., 2018), thus representing a more realistic description of the potential action of natural selection on the human genome. For this purpose, we computed the nSL statistics on whole genome sequence data generated by the 1000 Genomes Project for Han Chinese (CHB) and Japanese (JPT) populations (1000 Genomes Project Consortium et al., 2015) and we submitted the obtained genome‐wide distributions to the gene network algorithm implemented in the *signet* R package (Gouy, Daub, & Excoffier, 2017), as detailed in Gnecchi‐Ruscone et al. (2018). Assignment of genes to the related functional pathways was performed according to the Kyoto Encyclopedia of Genes and Genomes (KEGG) database and significant shifts (*p* < .05) toward extreme *signet* values were searched for in the distribution of scores observed within annotated pathways to identified gene networks pervasively subjected to positive selection. Networks of candidate adaptive genes were finally plotted using Cytoscape v3.6.0 (Shannon et al., 2003).

## 3 RESULTS

### 3.1 Exploring population structure in the assembled Pan‐Asian dataset

By applying stringent QC procedures to the generated genome‐wide data and by merging them with publicly available genotypes (see Methods), we obtained an “extended” dataset of 4,356 unrelated samples belonging to 162 worldwide populations characterized for 231,947 SNPs. When we submitted it to PCA, we observed the expected cline “V‐shaped” distribution of samples that reflects the main differentiation between populations of sub‐Saharan African ancestry (e.g., Esan and Yoruba from Nigeria, Bantu, Mbuti, and Biaka hunter‐gatherers, Mandenka, Luhya from Kenya) and non‐African groups (Figure S1), as pointed out by previous studies (Li et al., 2008; Mallick et al., 2016).

**FIGURE 1:**
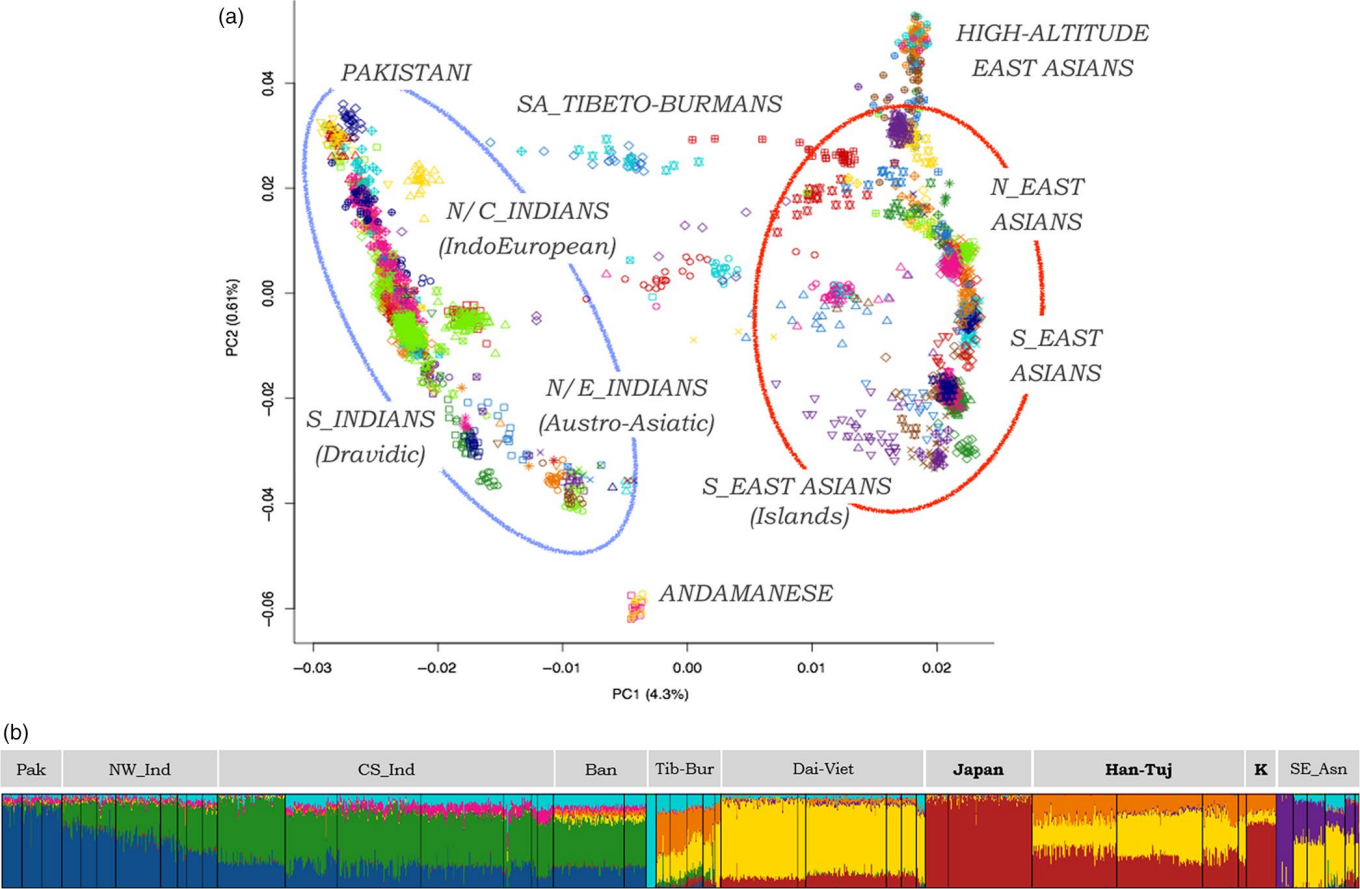
Principal component and ADMIXTURE analyses performed on the “Pan‐Asian” dataset. (a) PCA was applied to 2,379 individuals belonging to 124 populations from South and East Asia. Plot of PC1 versus PC2 pointed out the distinction between populations from the Indian subcontinent (blue ellipse) and groups from East Asian/South East Asian regions (red ellipse), along with gradients of genetic variation within both of these macro‐geographical areas. Individuals are color‐coded according to their population of origin, as described in the legend of Figure S2. (b) Results of ADMIXTURE clustering analysis at *K* = 8 performed on a refined dataset of 1,171 samples from 57 populations representative of the gradients of South Asian and East Asian variation (see Figures S3 and S4, for the full set of Ks tested and CV‐errors). Labels on top of the graph represent the main clusters identified: Pak, Pakistani; NW_Ind, North West Indians; CS_Ind, Central South Indians; Ban, Bangladeshis; Tib‐Bur, Tibeto‐Bumans; Dai‐Viet, Dai‐Vietnamese; Japan, Japanese; Han‐Tuj, Han‐Tujia Chinese; K, Koreans cluster; SE_Asn, South East Asians. In bold are reported the populations whose ancestors inhabited the regions where wild rice/millet originated and were early domesticated

To focus on the overall genomic landscape of long‐term cereal‐eating populations, we repeated PCA by retaining a “Pan‐Asian” subset of 2,379 individuals belonging to 124 populations from South and East Asia. Accordingly, PC1 was found to explain 4.3% of the investigated genomic variation, enabling to distinguish between populations from the Indian subcontinent and groups from East Asian/South East Asian regions (Figure 1a and Figure S2). PC2 instead accounted for 0.61% of variance and described two latitudinal clines of diversity within both of these macro‐geographical areas. On one side, South Asian populations ranged from Pakistani ethnic groups and Northern/Central Indian people speaking Indo‐European languages to Southern Indian tribes speaking Dravidian languages and Austro‐Asiatic speaking populations from Northeast India (Figure 1a), as previously described (Basu, Sarkar‐Roy, & Majumder, 2016; Metspalu et al., 2011; Reich, Thangaraj, Patterson, Price, & Singh, 2009). On the other side, an East Asian gradient of variation was delimited by the opposite ends represented by high‐altitude Himalayan groups and the Malay and other Austronesian populations (Figure 1a), reflecting the occurrence of complex demographic and adaptive processes more than a simple distribution along a latitudinal cline, as suggested by extensive literature (HUGO Pan‐Asian SNP Consortium, 2009; Gnecchi‐Ruscone et al., 2017, 2018). Uygurs, Hazaras, and groups from Northeast India and Nepal that speak Tibeto‐Burman languages instead occupied an intermediate position in the PCA space between the two previously described clines of variation (Figure 1a) in accordance with the admixture events they have experienced in historical times (Gnecchi‐Ruscone et al., 2017; Li et al., 2008). Finally, individuals from the Andaman and Nicobar Islands and belonging to the Onge and Jarawa populations represented genetic outliers with respect to the bulk of the examined South Asian and East Asian groups mainly due to their shared ancestry with Oceanic Pacific Islanders and prolonged isolation, as already proposed by Basu et al. (2016).

### 3.2 Identification of genetically homogeneous population clusters

Principal component analysis results were used to refine the assembled “Pan‐Asian” dataset by removing populations representing unusual outliers within the considered genomic landscape. We thus selected 1,171 samples belonging to 57 populations well representative of the described gradients of South Asian and East Asian variation and we submitted them to ADMIXTURE analyses to identify population clusters showing high internal genetic homogeneity and thus having presumably shared a common genetic history. We tested *K* = 2 to *K* = 10 potential ancestral groups (Supplementary Results and Figure S3) and the best predictive accuracy was achieved by the model when eight ancestry components (*K* = 8) were assumed (Figure S4). The overall picture of genetic components recognized within each population at K = 8 (Figure 1b) was concordant with patterns of population structure pointed out by PCA and with findings from previous studies. In particular, Pakistani people were found to be almost entirely characterized by the “Ancestral North Indian” component already identified by Reich et al. (2009), which was highly represented also in Indian Indo‐European speaking populations, while an “Ancestral South Indian” component was predominant in Indian groups speaking Dravidian languages (Metspalu et al., 2011) (Figure 1b). Two additional ancestry fractions characterized Indian Austro‐Asiatic speaking tribes and speakers of Tibeto‐Burman languages (Figure 1b), as previously observed by Basu et al. (2016) and Gnecchi‐Ruscone et al. (2017). Some Tibeto‐Burman groups (e.g., Burmese and Lahu) also showed a minor proportion of the genome ascribable to the “Ancestral South Indian” component, which was evident even in the Malays (Figure 1b) and pointed to a reduced Indian gene flow to South East Asia in contrast to a documented cultural influence since at least 2,500 years ago (Mörseburg et al., 2016). A further component was found to be associated mainly to northern East Asian populations, representing a unique genetic signature for Japanese people and reaching high percentages in Koreans. On the contrary, a different component appeared to be related to southern East Asian populations, being overwhelming among Cambodian, Dai, Lahu, Vietnamese, and Austronesian groups. A specific ancestry fraction plausibly associated to the expansion of Austronesian languages and the related demographic processes (Mörseburg et al., 2016) was observed to be predominant in the Igorots and was significantly represented in the Dusuns and Muruts from Borneo, as well as in the Malays and some Filipino groups (e.g., Luz and Vizaya) (Figure 1b). Finally, Birhor people showed an almost fixed ancestry component, which was widespread, although at low percentages, also in all South Asian and in many South East Asian populations (Figure 1b).

**FIGURE 2:**
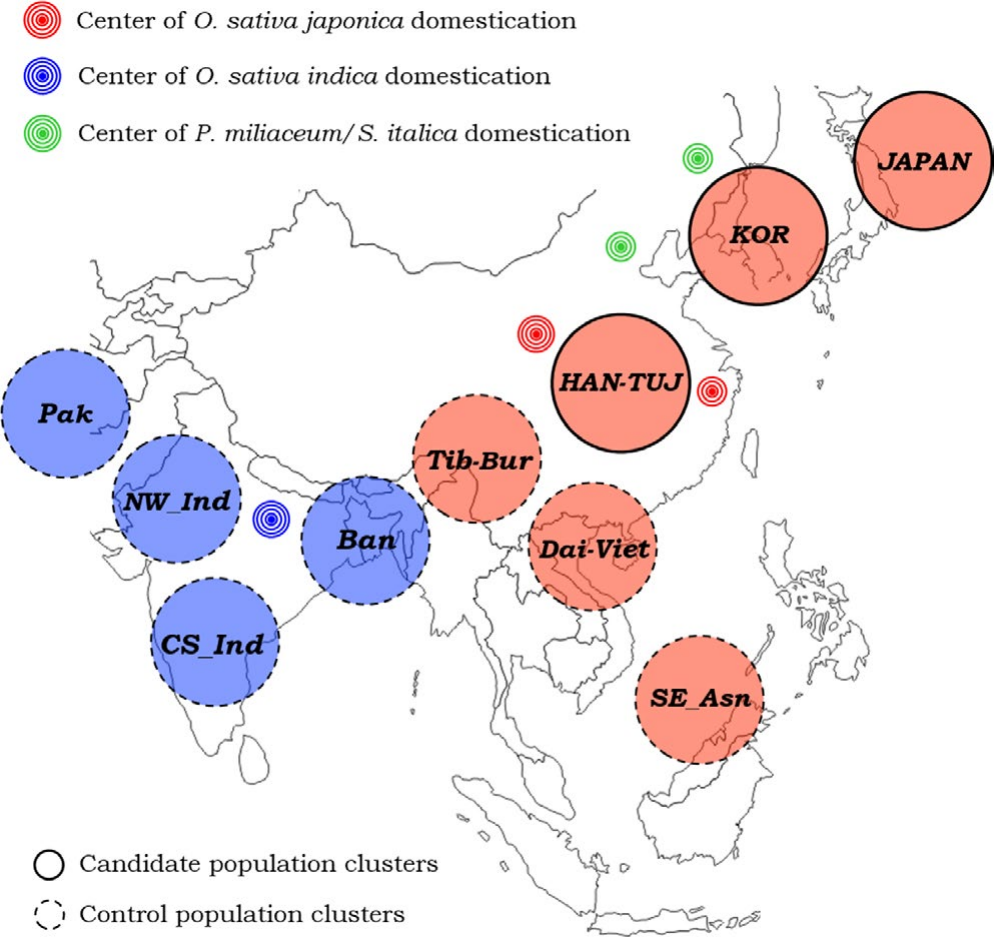
Approximate geographical distribution of Asian population clusters pointed out by ADMIXTURE analysis and their relative position with respect to known centers of rice/millet domestication. Blue clusters showed predominant South Asian ancestry, while red ones are enriched for East and South East Asian ancestry. Labels of each cluster are reported as described in the legend of Figure 1b. Red concentric circles indicate archaeological sites along the Yangtze River valley in Eastern China where remains suggesting usual consumption of wild rice have been dated to at least 12,000 years ago and where *O. sativa japonica* was early domesticated. From there, rice agriculture diffused primarily to the Korean peninsula and the Japanese archipelago. Green concentric circles indicate archaeological sites in the Hebei and Manchuria provinces of Northern China where remains suggesting early cultivation of broomcorn millet and foxtail millet were found. Populations native from these and the nearby regions thus represent the candidate clusters tested for adaptations to cereal‐based diets and are highlighted by bold circles. Conversely, all the remaining clusters were used as control groups (i.e., populations not expected to have evolved adaptations to cereal‐based diets despite using rice as a staple food). Blue concentric circles indicate archaeological sites across the Indo‐Gangetic Plain where evidence for more recent domestication of *O. sativa indica* was found. Such a domestication process was likely enabled by hybridization of *O. sativa japonica* from China with the local proto‐indica rice

Distribution of ancestry components depicted by ADMITXUTRE analysis at *K* = 8 enabled us to identify 10 different population clusters characterized by high internal genetic homogeneity (i.e., made up by individuals presenting the same ancestry components admixed at approximately the same proportions) and that grouped together populations showing also close geographical proximity, similar cultural patterns, and largely shared demographic histories (Figure 2). In particular, we identified the *Pakistani cluster* (Makrani, Balochi, and Brahui individuals; *N* = 74); the *North West Indian cluster* (Pathan, Khatri, Sindhi, Punjabi people, and Gujarati Brahmins; *N* = 141); the *Central South Indian cluster* (Gujarati people, inhabitants of the Uttar Pradesh, Telegu, and other speakers of Dravidian languages, Sri Lankan Tamil, Maratha, and Pallan castes; *N* = 410); the *Bangladeshi* cluster (*N* = 114); the *Tibeto‐Buman cluster* (Jamatia, Tripuri, Lahu and people from Myanmar; *N* = 80); the *Dai‐Vietnamese cluster* (*N* = 239); the *Japanese cluster* (*N* = 132); the *Han‐Tujia cluster* (*N* = 262); the *Korean cluster* (*N* = 36); and the *South East Asian cluster* (Malays, Murut, Dusun, Vizaya, and Luz; *N* = 101).

### 3.3 Detection of genomic regions enriched for selective sweeps in each population cluster

By assuming that the ancestors of populations belonging to the same cluster have been plausibly subjected to similar selective pressures due to their largely shared environmental and cultural settings, we calculated genome‐wide distributions of the nSL statistics for each group of populations to detect the main selective sweeps having mediated their adaptive histories. To minimize false‐positive results, we searched for the most plausible candidate chromosomal intervals to have undergone positive selection by retaining top 1% genomic regions in the distribution of 200 kb windows ranked according to their proportion of outlier SNPs showing unusual nSL scores. We finally investigated functional relationships among genes laying in these top 1% windows according to information retrieved from the STRING protein–protein interactions database (see Methods).

Large genomic intervals enriched for signatures of positive selection thus turned out to be differentiated especially between clusters from the Indian subcontinent or South East Asia and groups from the northernmost East Asian regions covered by the dataset.

In particular, all South Asian clusters presented candidate adaptive genes that play a role in the ubiquitination process (Figure S5‐S9), a reversible post‐translational modification of cellular proteins able to regulate a broad set of mechanisms including cell division, differentiation, signal transduction, and protein trafficking (Mukhopadhyay & Riezman, 2007). In detail, the *FBXW2* ring finger proteins class (RNF), *LTN1*, *SPSB4*, *ZBTB16,* and ubiquitin‐conjugating enzyme class (UBE) genes were some of the several targets of selection shared among the majority of South Asian and South East Asian clusters (Figure S5‐S10). The Pakistani, Tibeto‐Buman, South East Asian, and Dai‐Vietnamese groups also shared selection signatures on many genes functionally related to the *PIK3CA* locus (Figure S1, S9‐S11). This contributes to the activation of signaling cascades involved in cell growth, survival, proliferation, motility, and morphology, indirectly through the generation of phosphatidylinositol 3,4,5 trisphosphate. Moreover, it participates in cellular signaling in response to various growth factors and in the promotion of developmental processes in diverse tissues (Yamaguchi et al., 2011). Among loci functionally related to *PIK3CA*, *ERBB4* is known to contribute to regulation of the development of heart, central nervous system, and mammary gland, as well as to specific gene transcription, cell proliferation, differentiation, migration, and apoptosis (Iwamoto & Mekada, 2006; Muraoka‐Cook et al., 2006; Sweeney et al., 2000). *BLK* instead encodes for a nonreceptor tyrosine kinase involved in B‐lymphocyte development, differentiation, and signaling (Borowiec et al., 2009). Finally, *EPHB1* is responsible of cell migration and adhesion, especially during the nervous system development, when it regulates retinal axon guidance.

Selection signatures at genomic intervals associated to completely different biological functions with respect to those described above were instead observed for the Han‐Tujia, Japanese, and Korean clusters. The highest number of genes belonging to the top 1% genomic windows putatively subjected to selection and functionally related to each other (i.e., involved primarily in fat tissue metabolism) was observed in people of Han or Tujia ancestry (Figure S12). For instance, *AKR1C2* is a member of the aldo/keto reductase superfamily that catalyzes the conversion of aldehydes and ketones into their corresponding alcohols. It is mainly expressed in fat tissue (Fagerberg et al., 2014) and bind bile acid with high affinity to convert steroid hormones into the 3‐alpha/5‐alpha and 3‐alpha/5‐beta‐tetrahydrosteroids (Couture et al., 2005; Faucher et al., 2007; Hara et al., 1996). The *ALDH9A1* gene instead encodes for an enzyme that belongs to the aldehyde dehydrogenase protein family and has a high activity for oxidation of gamma‐aminobutyraldehyde and other amino aldehydes (Vaz, Fouchier, Ofman, Sommer, & Wanders, 2000). Likewise *AKR1C2*, it is expressed mainly in fat tissue (Fagerberg et al., 2014). The *ACSF2* locus codes for a protein that catalyzes the initial reaction in fatty acid metabolism and plays a role in adipocyte differentiation (Watkins, Maiguel, Jia, & Pevsner, 2007). *CYP1B1* is a member of the cytochrome P450 superfamily of enzymes, monooxygenases that catalyze many reactions involved in drug metabolism and synthesis of cholesterol, steroids, retinoids, and xenobiotics (Shimada et al., 1999). *ACSL3* encodes for an isozyme of the long‐chain fatty‐acid‐coenzyme A ligase family that converts free long‐chain fatty acids into fatty acyl‐CoA esters and thereby play a key role in lipid biosynthesis and fatty acid degradation (Yao & Ye, 2008). *ABHD5* codes for a lysophosphatidic acid acyltransferase that contributes to phosphatidic acid biosynthesis. Mutations at this gene have been associated with Chanarin‐Dorfman syndrome, a triglyceride storage disease with impaired long‐chain fatty acid oxidation (Ghosh, Ramakrishnan, Chandramohan, & Rajasekharan, 2008). Finally, *GPX3* and *MGST3* code for proteins that protect cells and enzymes from oxidative damage by catalyzing the reduction of hydrogen peroxide, lipid peroxides, and organic hydro peroxide.

Putative adaptive genes that showed functional relationships with each other were identified also for the Japanese cluster and involved again *ALDH9A1*, *GPX3,* and *MGST3* (Figure S13). Moreover, a beta‐adducin (*ADD2*), a TNFAIP3‐interacting protein 3 (*TNIP3*), whose overexpression inhibits NF‐kappa B‐dependent gene expression in response to lipopolysaccharide (Wullaert et al., 2007), as well as a chromodomain Y‐like protein (*CDYL*), a glyoxylate reductase 1 homolog (*GLYR1*) and *ADH7* (alcohol dehydrogenase 7) were found to participate to the same biological processes. Even if it is a member of the alcohol dehydrogenase family, the enzyme encoded by *ADH7* is most active as a retinol dehydrogenase. Its expression is greatly concentrated in the esophagus (Fagerberg et al., 2014), while being almost absent in other tissues, and its variants have been linked to alcohol dependence (Park et al., 2013) and oral cavity or pharyngeal cancer (McKay et al., 2011).

Some of these genes were pointed out as candidate targets of positive selection also in the Korean cluster (Figure S14), as is the case of *ALDH9A1*, *ACSF2,* and *ADD2*, which were previously observed in the Han‐Tujia and Japanese clusters, respectively. However, in people of Korean ancestry, these loci were found to be functionally related also to two additional candidate adaptive genes (i.e., *ALDH1A2* and *AOX1*). The *ALDH1A2* locus encodes for an enzyme that catalyzes the synthesis of retinoic acid from retinaldehyde. Similarly to *ADH7*, it has been associated to Barrett's esophagus, a premalignant precursor of esophageal adenocarcinoma (Gharahkhani et al., 2016; Levine et al., 2013). The *AOX1* gene codes instead for an oxidase with broad substrate specificity (Beedham, Critchley, & Rance, 1995). Furthermore, the Korean cluster presented a second large signature ascribable to genes relevant in the lipid metabolism (Figure S14). It included acetyl and acyl transferases (*NAA20*, *AGAPAT1,* and *MOGAT1*) and *LPIN1*, which play important roles in controlling fatty acids metabolism at different levels.

### 3.4 Fine mapping of the most informative candidate adaptive genes

After having identified large genomic regions plausibly subjected to positive selection, along with the associated biological functions, we prioritized the genes contained in them (see Methods) and we focused on the loci showing the strongest selective signatures in each cluster.

Again, we observed appreciably different adaptive profiles among the considered clusters, especially between South Asian/South East Asian groups and East Asian people from more northerly latitudes. In fact, only four genes (i.e., *DENND1A*, *MCC*, *SCRN1,* and *WIPF3*) were found to be included in the top 1% windows of all clusters (Table S2). Among them, *DENND1A* showed the strongest and widest signature of selection as its variants were generally included in chromosomal intervals characterized by the highest‐ranking positions in the majority of clusters and were distributed through different windows. Several genome‐wide association studies suggested that variation at this gene likely influences polycystic ovary syndrome risk and succeeded in identifying risk SNPs that showed remarkable association with the disease in most populations of Asian ancestry (Chen et al., 2011; Ha, Shi, Zhao, Li, & Chen, 2015; Shi et al., 2012). In addition, the Pakistani and the North West Indian clusters shared five candidate adaptive genes (i.e., *FBXW2*, *LOC253039*, *PHF19*, *TRAF1‐C5,* and *NRG3*), with a limited overlap also with signatures observed in the Bangladeshi group (i.e., *FBXW2* and *PHF19*).

Conversely, the remaining genes included in the top 1% candidate windows were found to be differentiated among most of the examined clusters (Table S2). Interestingly, similar results were pointed out for the Han‐Tujia and Japanese groups, which distinguished them also from most of the other East Asian clusters. In fact, they shared significant nSL scores at SNPs located in a wide genomic region including the *ERGIC3*, *FER1L4,* and *CPNE1* genes. Among them, *ERGIC3* represented the most interesting locus because genome‐wide association studies have supported its implication in the modulation of total cholesterol levels (Teslovich et al., 2010; Willer et al., 2013). In particular, the derived allele (T) of rs2277862 was reported to significantly correlate with lower level of such a lipid trait and to present *cis*‐acting associations with *ERGIC3* transcript levels in liver, omental, and subcutaneous fat (Teslovich et al., 2010). We found this allele to be encompassed in the genomic window showing one of the strongest signatures of positive selection in both the Han‐Tujia and Japanese clusters, being also included in the top 1% windows identified in the Korean group. This region also harbors other six and seven SNPs presenting unusual nSL values respectively in the Han‐Tujia and Japanese populations. Moreover, with the sole exception of rs6088887, which was found to be an outlier SNP only in people of Han or Tujia ancestry, exactly the same variants were pointed out as potential targets of selection in both the Han‐Tujia and Japanese clusters.

Finally, *FOXP1* was included in the top 1% windows of both Han‐Tujia, Japanese, and Korean groups, but emerged among the top 10 candidate genes only in the Japanese population.

This gene belongs to the FOX family of transcription factors and is involved in a broad range of biological functions. It is indeed widely expressed and plays a role in embryological, immunological, and hematological processes, as well as in the development of speech and language (Benayoun, Caburet, & Veitia, 2011; Hannenhalli & Kaestner, 2009; Shi et al., 2008). Interestingly, in the context of a complex network of transcriptional factors and cofactors, it is also known to modulate hepatic glucose production in response to hormonal and nutrient stimuli (Nakae et al., 2002). In particular, we found several *FOXP1* variants contributing to one of the strongest signatures of selection observed in the Han‐Tujia, Japanese, and Korean clusters.

### 3.5 Investigation of polygenic adaptive events in the Han‐Tujia and Japanese clusters

Both windows‐based and single gene level nSL analyses pointed to the Han‐Tujia, Korean, and Japanese clusters as the groups having most plausibly evolved adaptations triggered by dietary‐related selective pressures. To validate these findings on independent cohorts of samples and to deepen the investigation of their adaptive history, we used whole genome sequence data generated by the 1000 Genomes Project (1000 Genomes Project Consortium et al., 2015) for the CHB and JPT populations and a gene network approach to test for the occurrence of adaptive events also according to a polygenic adaptation model (see Methods).

**FIGURE 3:**
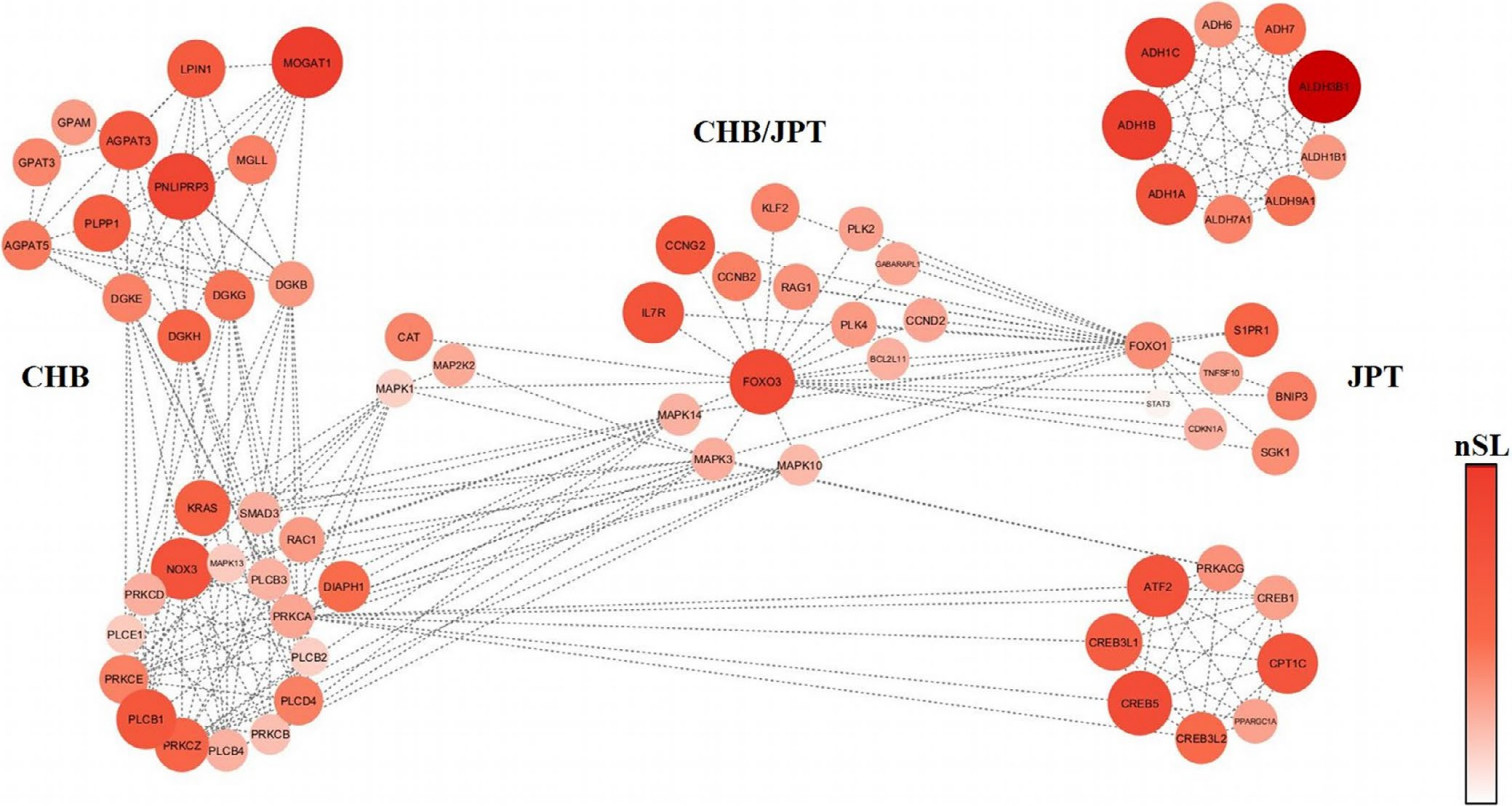
Gene networks showing signatures of pervasive positive selection in Han Chinese and/or Japanese populations according to *signet* analysis performed on whole genome distribution of nSL scores and aimed at testing for polygenic adaptation. On the left, genes belonging to the *Glycerolipid metabolism*, *AGE‐RAGE signaling in diabetic complications,* and *FoxO signaling* pathways pinpointed as candidate adaptive loci in the CHB population. On the right, genes belonging to the *FoxO signaling*, *Glycolysis/Gluconeogenesis,* and *Glucagon signaling* pathways pinpointed as candidate adaptive loci in the JPT population. In the center of the plot are reported the genes belonging to the *FoxO signaling* pathway that showed signatures of positive selection in both the considered populations. The size and color of the circles are proportional to the nSL peak value associated to each gene according to the displayed color‐scale

Gene networks belonging to the *Glycerolipid metabolism*, *AGE‐RAGE signaling in diabetic complications,* and *FoxO signaling* pathways were found to have undergone extensive positive selection in CHB (Figure 3, Table S3). Signatures at genes of the *Glycerolipid metabolism* pathway and involved in fatty acids metabolism, such as *LPIN1* and *MOGAT1*, were found to overlap with those detected by the windows‐based selection scan performed on the Korean cluster. Most of the remaining loci instead belonged to the other two pathways and have been implicated in the modulation of pathological traits associated to insulin resistance and T2D, as is the case of several genes encoding for mitogen‐activated proteins kinases (*MAPK*) and phospholipases (*PLCB*), or in the response to dietary stimuli, such as the insulin‐like growth factor 1 receptor (*IGF1R*) and *FOXO* genes (Figure 3, Table S3).

Gene networks belonging to the *FoxO signaling*, *Glycolysis/Gluconeogenesis,* and *Glucagon signaling* pathways were found to have been subjected to positive selection in JPT (Figure 3, Table S4). In details, multiple *MAPK* and *FOXO* genes were the same pointed out as candidate loci in CHB, while *ALDH9A1* and *ADH7* were suggested to have undergone selective sweeps by single gene level analyses performed on the Han‐Tujia, Japanese, and Korean clusters. On the contrary, genes belonging to the *Glucagon signaling* pathway, such as several cAMP responsive element binding proteins (*CREB*), *PPARGC1A,* and *PRKACG*, were found to have played a putative adaptive role only in JPT (Figure 3, Table S4).

## 4 DISCUSSION

Introduction of massive cereals consumption, in particular of rice, in the diet of the ancestors of Asian populations has represented a substantial challenge for their metabolism due to the outstanding carbohydrates content and glycemic index of such a nutritional resource with respect to other domesticated plants (Atkinson et al., 2008). Daily and abundant rice intake is indeed considered a risk factor for the development of insulin resistance, thus potentially increasing susceptibility to T2D and obesity in populations that use it as a staple food (Boers et al., 2015; Hu et al., 2012). Nevertheless, epidemiological patterns related to these metabolic diseases present appreciable variation across human groups of Asian ancestry. In fact, with respect to people from the Indian subcontinent, some East Asian populations show a lower increase in T2D prevalence subsequent to the shifts in dietary habits and lifestyles due to the social and economic transitions occurred in most Asian regions in the last decades (Chan et al., 2009; Ramachandran et al., 2010; Yoon et al., 2006). Accordingly, we hypothesized that genetic adaptations against the detrimental side effects of cereal‐based diets evolved by populations that relied on these nutritional resources for a period long enough to enable natural selection to shape variation at disease‐associated loci might contribute to such differential metabolic risk. In particular, human groups from Eastern China, whose ancestors have routinely consumed wild rice long before (i.e., since around 10,000 years ago) its domestication and the development of cultivation techniques (Cao et al., 2006; Jiang & Liu, 2006; Zhao, 1998), represent the most reliable candidate populations to be tested for the occurrence of these adaptive events. Secondly, people from Korea and Japan, the regions to which millet and rice agriculture early diffused from China (Gross & Zhao, 2014), may have evolved comparable adaptive traits, contrary to populations of South East Asia or the Indian subcontinent who instead adopted usual cereals consumption only several thousand years later (Choi et al., 2017; Gross & Zhao, 2014; Molina et al., 2011).

To test this hypothesis, we investigated the adaptive evolution of populations whose ancestors inhabited the geographical areas where wild rice and/or millet originated and were early domesticated (i.e., Han and Tujia Chinese groups, Koreans, and Japanese people) and compared it with that of control groups from South East Asian and South Asian regions where cereals agriculture spread only more recently. For this purpose, we took advantage from newly generated and literature genomic data for 2,379 individuals belonging to 124 Asian populations.

### 4.1 Population clusters within the South Asian and East Asian genomic landscapes

To frame patterns of genetic diversity observable at both candidate and control populations within the overall South Asian and East Asian genomic landscape and to test for representativeness of the selected samples with regard to their ethnic group, we applied PCA and ADMIXTURE algorithms to the assembled “Pan‐Asian” dataset. These population structure analyses confirmed the well‐known genetic differentiation between groups from the Indian subcontinent and from East Asian or South East Asian regions, along with gradients of variation observable within these macro‐geographical areas (Figure 1a).

In detail, genetic affinity among populations of South Asian ancestry was found to follow a cline that roughly reflects both their latitudinal dislocation across the Indian subcontinent and the distribution of different language groups, as previously attested by several studies (Basu et al., 2016; Chaubey et al., 2011; Majumder, 2010; Metspalu et al., 2011; Reich et al., 2009). People speaking Indo‐European languages and showing high proportions of the “Ancestral North Indian” ancestry component and groups speaking Dravidian languages, who are instead characterized by the “Ancestral South Indian” component, were located at the opposite ends of the South Asian gradient of diversity (Figure 1a). On the contrary, tribes speaking Austro‐Asiatic languages and presenting the “Ancestral Austro‐Asiatic” ancestry component (Figure 1b) were more scattered in the PCA space, with especially those from Eastern India clustering close to Dravidian populations and being slightly shifted toward the East Asian cline (Figure 1a). As expected, both PCA and ADMIXTURE analyses pointed to populations characterized by the “Ancestral Tibeto‐Burman” component to be more genetically similar to East Asians than to South Asians, despite being located mainly south of the Himalayas (Figure 1). This confirms the findings from studies that indicated groups speaking Tibeto‐Burman languages as clear examples of gene flow from the Far East to the Indian subcontinent (Chaubey et al., 2011; Gnecchi‐Ruscone et al., 2017; Mörseburg et al., 2016).

A complex gradient of genetic diversity was observed when considering people of East Asian and South East Asian ancestries, plausibly reflecting the tangled patterns of admixture events, isolation processes and adaptive dynamics experienced by these ethnic groups. Tibetans and Sherpa stood out at the top of this cline of variation and resulted relatively differentiated from the bulk of East Asian peoples (Figure 1a). This has been proved to be ascribable to their prolonged isolation on high‐altitude Himalayan regions, which was made possible by the evolution of genetic adaptations to hypobaric hypoxia (Beall et al., 2010; Gnecchi‐Ruscone et al., 2018; Simonson et al., 2010; Yi et al., 2010). Long‐term isolation is also supposed to have driven the “Ancestral Tibeto‐Burman” ancestry component near to fixation especially in Sherpa groups (Figure 1b) due to strong genetic drift (Gnecchi‐Ruscone et al., 2017). Conversely, populations from the Malay Peninsula, Borneo, and Philippines were located at the opposite end of the East Asian/South East Asian cline of variation (Figure 1a). According to ADMIXTURE analyses, they showed remarkable proportions of an ancestry fraction that previous studies proposed to have been spread by demographic processes related to the expansion of Austronesian languages (Figure 1b) (Mörseburg et al., 2016).

Given that ADMIXTURE runs testing eight potential ancestral groups turned out to be the model that best fitted with the data (Figure S4), profiles of ancestry components depicted at K = 8 were used to guide the identification of distinguishable population clusters. Accordingly, 10 clusters characterized by high internal genetic homogeneity and grouping populations with close geographical proximity, similar cultural patterns, and shared demographic histories were described (Figure 2) and used to maximize the number of samples to be submitted to selection scans. Populations of Han‐Tujia, Korean, and Japanese ancestries, whose archaeobotanical evidence suggest to be the sole groups having relied on rice/millet consumption long before their cultivation or in conjunction with their very ancient domestication (Fuller et al., 2010; Gross & Zhao, 2014; Zhao, 1998), represented the best candidate clusters for testing the evolution of adaptive events triggered by cereal‐based diets. Conversely, the remaining clusters were considered as control groups who have adopted such a typology of diet too recently to genetically adapt to it. For instance, the earliest evidence of usual rice consumption outside Eastern China, Korea, and Japan was associated to villages with domestic rice livestock located in the Indo‐Gangetic Plain, which have been proved to become widely established not earlier than 4,000 years ago (Fuller, 2006; Fuller et al., 2010).

### 4.2 Distinctive patterns of adaptive evolution in candidate and control population clusters

Large chromosomal intervals enriched for footprints left by the action of positive selection were identified by means of a sliding windows approach based on the computation of genome‐wide distributions of nSL scores for each population clusters (Figure S5‐S14, Table S2). Interestingly, adaptive profiles depicted for the candidate and control population groups were found to be highly differentiated in terms of both broad biological functions and single genes having experienced selective sweeps. In particular, most of the adaptive events inferred for the majority of South Asian and South East Asian control clusters occurred at loci involved in ubiquitination or in the regulation of cell proliferation/differentiation, for instance in the neural tissue or in the immune system (Figure S5‐S10). These signatures seem to concern essential developmental processes but are unlikely ascribable to adaptive evolution in response to dietary‐related selective pressures, certainly not to the adoption of cereals consumption. Therefore, the discussion of these findings was beyond the scope of the present study. Even when the most remarkable footprints of positive selection at single genes were shortlisted for each cluster, limited overlap between adaptive events inferred in candidate and control groups was observed (Table S2). Moreover, the few loci presenting evidence of selection in both South Asian and East/South East Asian clusters again seemed to have been not evolved in response to metabolic stresses. For instance, the gene showing the strongest and widest selection signature (i.e., *DENND1A*) plays a role in the development of polycystic ovaries, anovulation, and hyperandrogenism that mainly characterize the polycystic ovary endocrine syndrome (Chen et al., 2011; Ha et al., 2015; Shi et al., 2012).

Conversely, despite presenting distinct genetic backgrounds according to ADMIXTURE analyses (Figure 1b) and being known to have experienced comparable but not identical demographic histories (Wang, Lu, Chung, & Xu, 2018), people of Han and Tujia ancestries, along with populations from Korea and Japan, showed adaptive profiles very similar with each other and remarkably different with respect to those of control clusters. It is worth noting that these candidate groups overall showed evidence of adaptive evolution at genes implicated in biochemical processes occurring in the fat tissue, in particular at the level of fatty acids metabolism, cholesterol and other lipids biosynthesis, and adipocyte differentiation (Fagerberg et al., 2014; Ghosh et al., 2008; Watkins et al., 2007; Yao & Ye, 2008). For instance, the *LPIN1* gene was suggested to have experienced a selective sweep in Koreans according to windows‐based nSL analyses (Figure S14) and to have contributed to polygenic adaptation of Han people according to the gene network approach applied to CHB whole genome sequence data (Figure 3, Table S3). This locus acts as a key regulator of fatty acids metabolism in several model organisms (Harris & Finck, 2011), and variants reducing its expression in the human adipose tissue have been associated with insulin resistance and impaired glucose homeostasis (Aulchenko et al., 2007; Bego et al., 2011; Loos et al., 2007; Suviolahti et al., 2006; Yao‐Borengasser et al., 2006; Zhang et al., 2013). In addition, the *ERGIC3* gene, which is involved in the modulation of cholesterol biosynthesis, has undergone selective sweeps in all the candidate clusters, showing a putative adaptive haplotype highly conserved especially between people of Han/Tujia and Japanese ancestries (Table S2). This haplotype carries derived alleles known to be associated to low cholesterol levels and reduced risk of cardiovascular and metabolic traits, including coronary artery disease, waist‐hip ratio, and BMI (Teslovich et al., 2010; Willer et al., 2013). This might suggest genetic adaptation resulting in the reduction of both the conversion of dietary carbohydrates into cholesterol and fatty acids and of the combination of the latter with glycerol to form triglycerides to be stored in the adipose tissue. Similarly, convergent selective sweeps at the *FOXP1* gene were inferred for both the Han‐Tujia, Korean, and Japanese clusters (Table S2), in addition to pervasive selection at multiple genes functionally related to this locus (e.g., *FOXO1*) as pointed out by gene‐network analyses performed on CHB and JPT genome sequences (Figure 3, Tables S3‐S4). *FOXO1* is involved also in the regulation of hepatic synthesis of glucose in response to hormonal and nutrient stimuli (Nakae et al., 2002) by promoting its production to prevent life‐threatening hypoglycemia during prolonged starvation (Matsumoto, Pocai, Rossetti, Depinho, & Accili, 2007). In particular, decreased blood insulin levels represent a signal for *FOXO1* to stimulate the expression of key gluconeogenic enzymes via direct binding to insulin response elements (IREs) located in their promoter regions, while *FOXP1* acts as a transcriptional repressor that inhibits them by directly interacting with the *FOXO1* gene or by competing with it to bind IREs of target genes (Accili & Arden, 2004; Zou et al., 2015). Interestingly, abnormal elevation of hepatic glucose production is known to contribute to fasting hyperglycemia in T2D (Saltiel & Kahn, 2001), and in a diabetic murine model increased gluconeogenesis promoted by *FOXO1* seems to be ascribable to decreased *FOXP1* expression in the liver. Accordingly, induced *FOXP1* overexpression has been proved to suppress hepatic gluconeogenesis in these diabetic mice, thus contributing to reduce glycemia and to re‐establish glucose homeostasis (Zou et al., 2015). Therefore, we can speculate that adaptive evolution at *FOXP1* and *FOXO1* genes might play a relevant role in the fine‐tuning of blood glucose levels in populations having long relied on a staple food characterized by medium‐to‐high glycemic index, such as rice or millet (Atkinson et al., 2008). An analogous explanation may be invoked also for selection signatures at loci belonging to the *Glucagon signaling* pathway identified by gene‐network analyses in people of Japanese ancestry (Figure 3, Table S4). Among them, the *CREB1*, *ATF2*, *PPARGC1A*, and *CPT1C* genes are indeed known to modulate insulin resistance in the adipose tissue of obese individuals (Gao et al., 2009; Maekawa, Jin, & Ishii, 2010; McCarty, 2005; Qi et al., 2009).

Finally, putative adaptive events shared by all candidate clusters were observed at loci encoding for alcohol and aldehyde dehydrogenases, particularly at genes (e.g., *ADH7*, *ALDH1A2,* and *ALDH9A1*) implicated in the oxidation of retinol to retinaldehyde and in the subsequent synthesis of retinoic acid from it (Figure 3, Figure S12‐S14, and Table S4). This pattern might reflect adaptations of Han, Tujia, Korean, and Japanese people aimed at optimizing the production of retinoic acid, which is the active metabolite of vitamin A and a powerful regulator of gene transcription that plays a crucial role in cellular proliferation and differentiation (Ross & Zolfaghari, 2011). In fact, vitamin A deficiency represents a major health issue for many developing societies (West & Darnton‐Hill, 2001), being especially associated in most Asian populations with the poverty‐related predominant consumption of rice, which lacks pro‐vitamin A in the edible part of the grain (Paine et al., 2005).

In conclusion, by using genome‐wide SNP and whole genome sequence data to reconstruct the adaptive history of a large “Pan‐Asian” set of populations, we provided evidence for selective sweeps and polygenic mechanisms having possibly contributed to the biological adaptation to rice‐ and/or millet‐based diets of people of Han/Tujia, Korean and Japanese ancestries. Actually, inferring the specific environmental or cultural stresses having triggered a given biological adaptation is not straightforward and we cannot rule out the possibility that other selective pressures in addition to those represented by cereal‐based diets have played a role in shaping the detected signatures of adaptive evolution. For instance, climate conditions differ between the geographical areas occupied by candidate and control populations, with temperate/continental climate being typical of northern China, northern Japan, and Korea in contrast to the subtropical or tropical climates of most Indian regions. Accordingly, some metabolic adaptations observed in the East Asian candidate populations might have been evolved also in response to a more energy demanding environment than that in which South Asian people live, as previously suggested for other adaptive loci (Sazzini et al., 2014). Indeed, these traits might be implicated in optimizing the use of high‐calories nutrients such as carbohydrates as fuel for thermogenic processes rather than as sources for triglycerides storing in the adipose tissue, thus resulting in better glucose homeostasis and reduced adiposity. Nevertheless, the described findings are reasonably consistent also with archaeobotanical data indicating that the ancestors of Han/Tujia, Korean, and Japanese populations have relied on rice or millet as a staple food since several thousand years before than other East Asian or South Asian populations did. In particular, our results suggested that the observed adaptations have been mediated by changes in fatty acids metabolism, in the biosynthesis of cholesterol and triglycerides from carbohydrates, as well as in the production of the vitamin A metabolite retinoic acid. Nowadays, these adaptive traits might play a role in mitigating some of the metabolic side effects of a usual massive intake of polished white rice, especially those related to the increased risk of developing overweight or obesity and impaired glucose homeostasis early in life, and in compensating the scarcity of vitamin A due to such a peculiar nutritional regimen. Therefore, the present study succeeded in pinpointing some of the deep causes that are contributing to influence present‐day epidemiological patterns observable across the Asian continent for T2D and obesity, thus taking a step forward in demonstrating the usefulness of an evolutionary approach to the study of the differential susceptibility of human populations to modern complex diseases.

## CONFLICT OF INTEREST

None declared.

## Supporting information

Supplementary Material

Table S1‐S2

## Data Availability

The genotype data generated during the current study are available at the corresponding author page of the figshare repository (https://figshare.com/authors/Marco_Sazzini/6292466).
