# Supplementary material for: Genomic adaptations to cereal‐based diets contribute to mitigate metabolic risk in some human populations of East Asian ancestry
Source: Evol Appl. 2020 Sep 8;14(2):297–313. doi: 10.1111/eva.13090 (PMC7896717; doi:10.1111/eva.13090)
Supplement: Supplementary file 1 — Supplementary Material [file EVA-14-297-s001.docx]

**Supporting Information**

Includes Figure S1-S14, Table S3-S4, and Supplementary Results.

Table S1-S2 are reported in a separate Excel file.

**Figure S1** PCA on the “extended” dataset made up of 4,356 individuals belonging to 162 populations from all over the world. Individuals are color-coded according to their population of origin as reported in the legend of the plot.

**
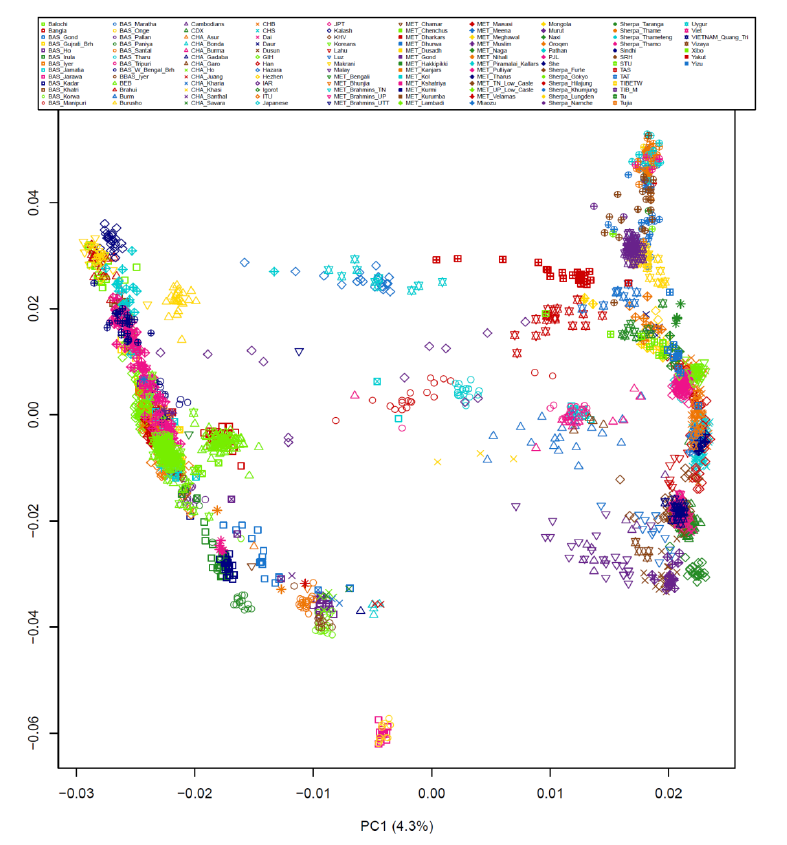
**

**Figure S2** PCA on the “Pan-Asian” dataset made up of 2,379 individuals belonging to 124 populations from South and East Asia. Individuals are color-coded according to their population of origin as reported in the legend of the plot.


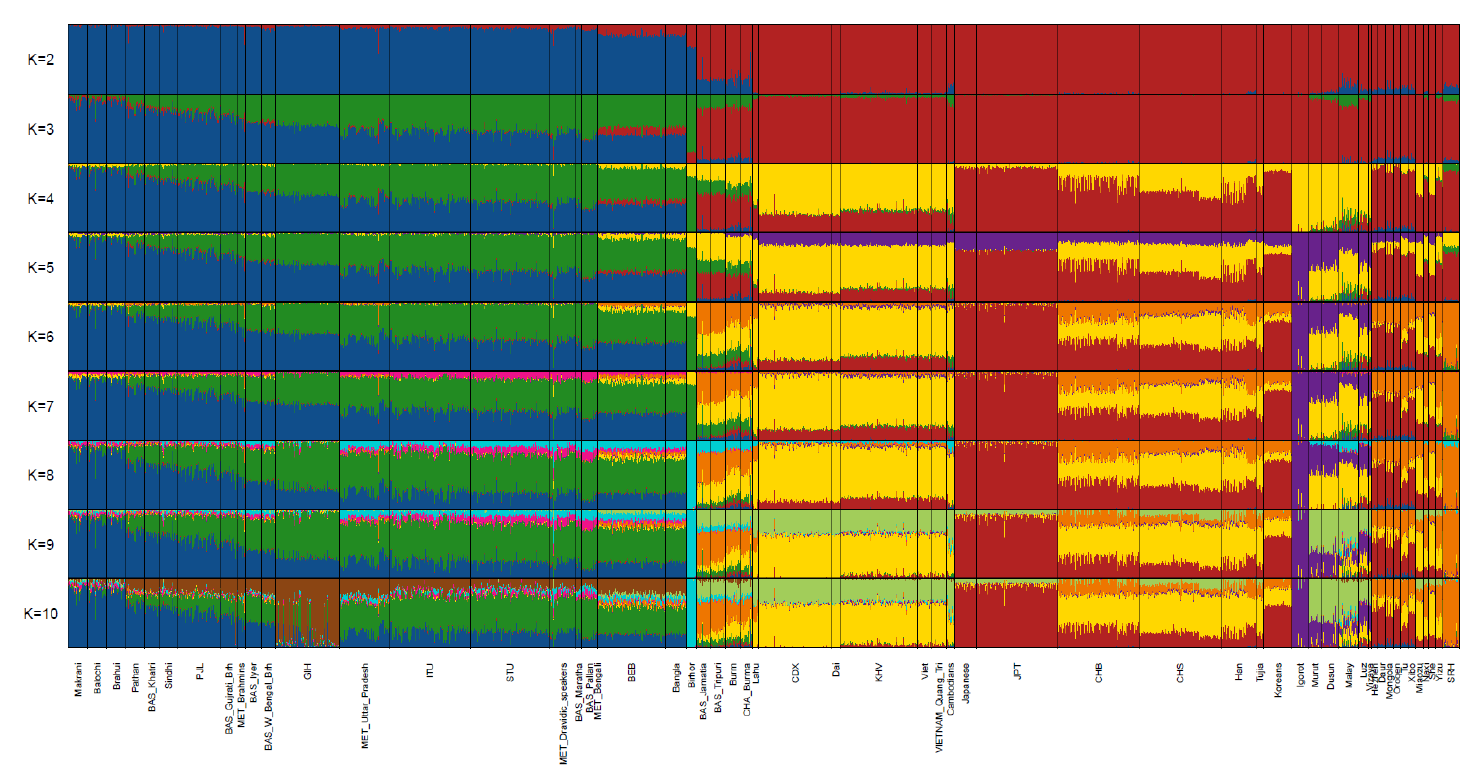


**Figure S3** ADMIXTURE analyses performed on a refined “Pan-Asian” dataset made up of 1,771 individuals belonging to 57 populations well representative of the gradients of South Asian and East Asian variation. Ancestry proportions were estimated by testing K = 2 (right) to K = 10 (left) potential ancestral populations. For each K only the runs showing the highest log-likelihood were plotted. Individuals are grouped and labelled at the population level.


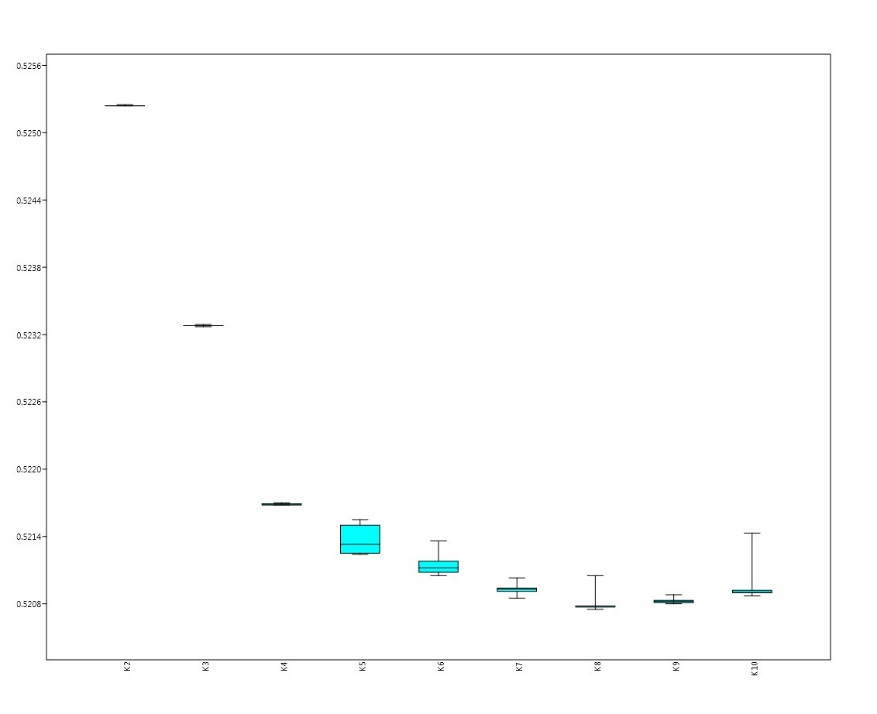


**Figure S4** Cross-validation (CV) errors for the ADMIXTURE clustering analyses reported in Figure S3. CV errors were computed for all the 50 independent runs performed for each of the tested K. The best predictive accuracy (i.e. lowest CV error) was achieved by the model when eight ancestral components (K = 8) were tested.

**
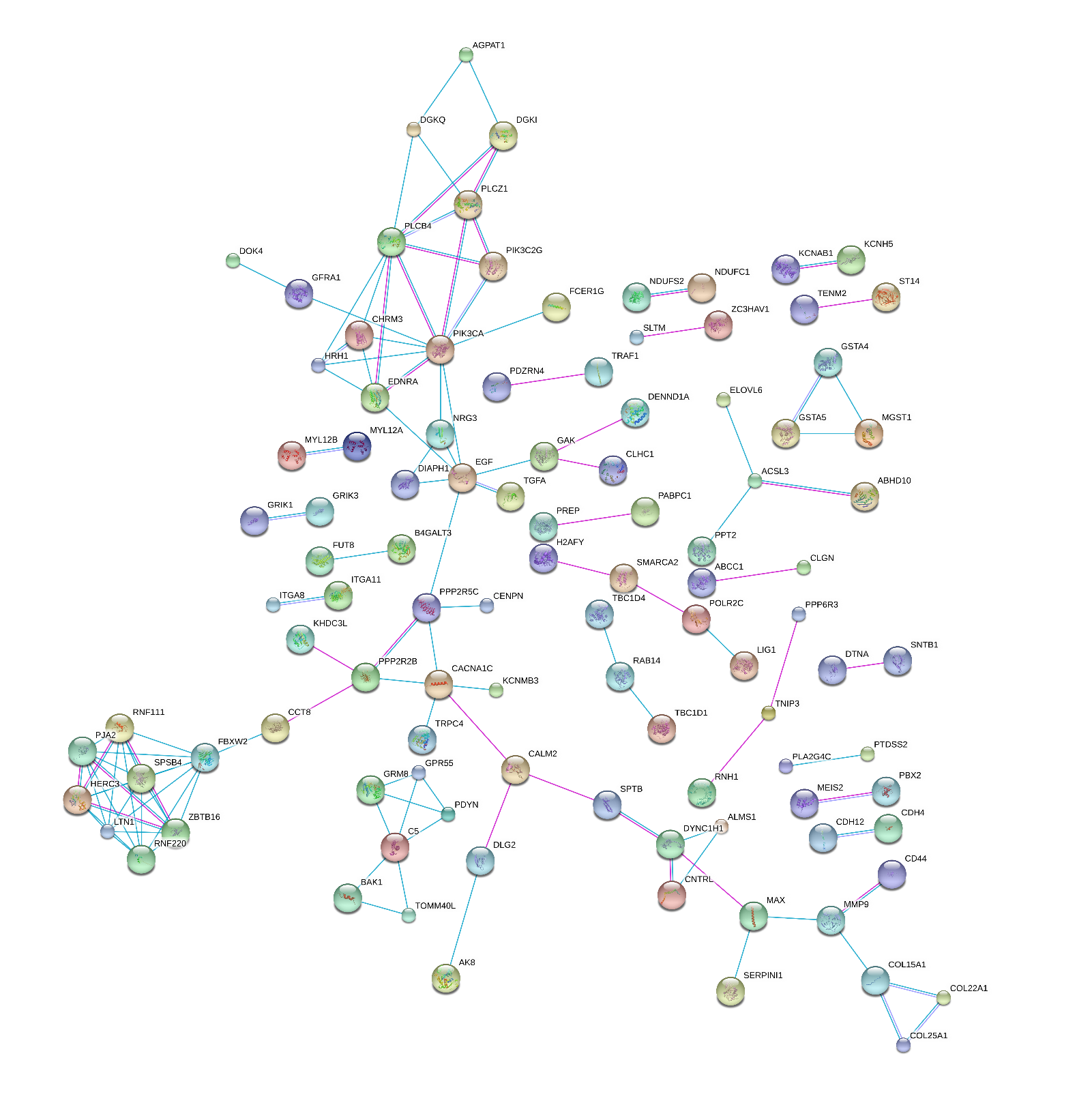
**

**Figure S5** Functionally related genes included the top 1% 200 kb windows ranked according to their proportion of outlier SNPs showing unusual nSL scores in the Pakistani cluster. Only loci related by known interactions annotated in the STRING protein-protein interactions database are considered. Interactions determined experimentally are displayed by pink edges, while those inferred from literature information are displayed by light blue edges.


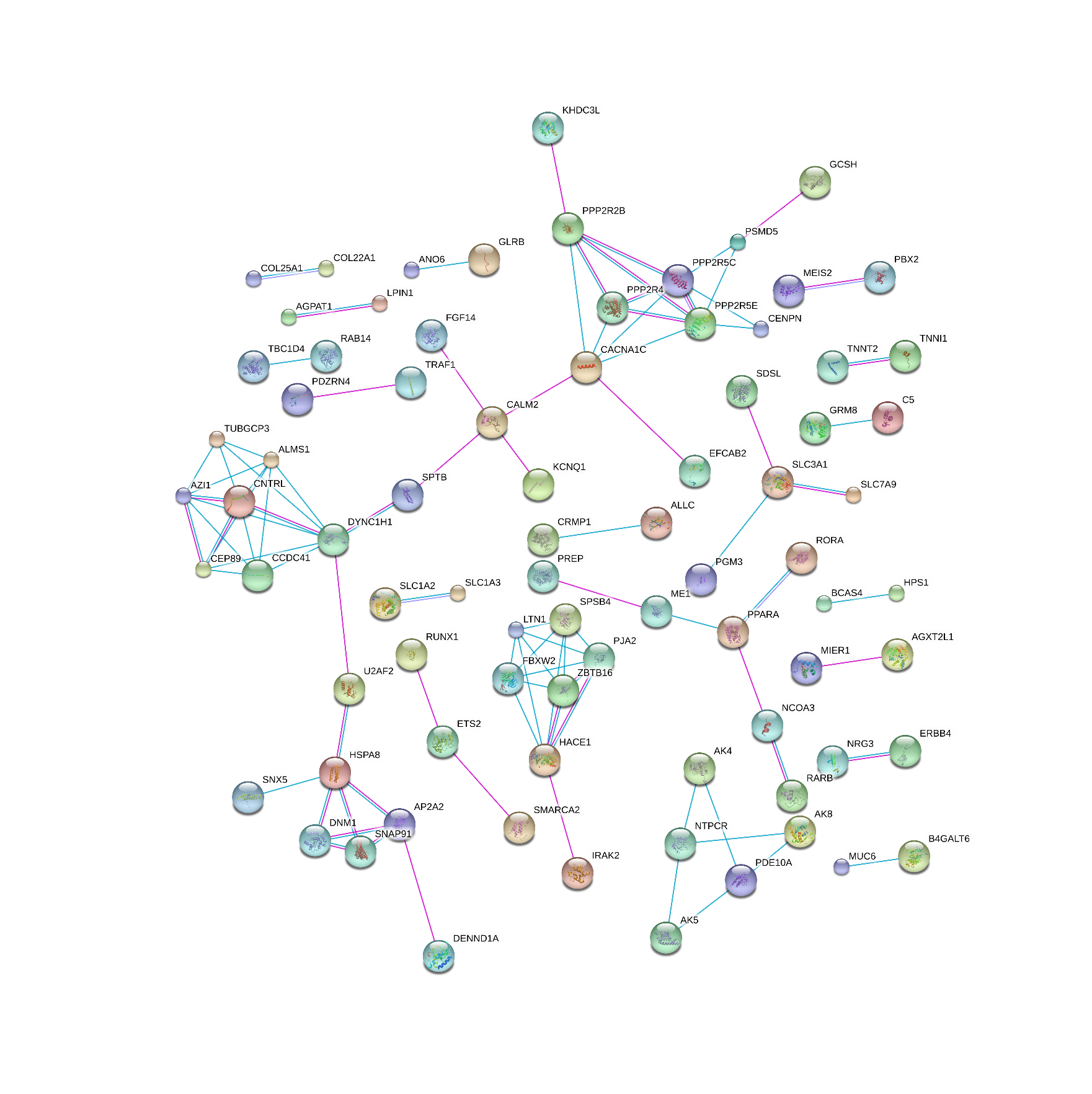


**Figure S6** Functionally related genes included the top 1% 200 kb windows ranked according to their proportion of outlier SNPs showing unusual nSL scores in the North West Indian cluster. Only loci related by known interactions annotated in the STRING protein-protein interactions database are considered. Interactions determined experimentally are displayed by pink edges, while those inferred from literature information are displayed by light blue edges.

**
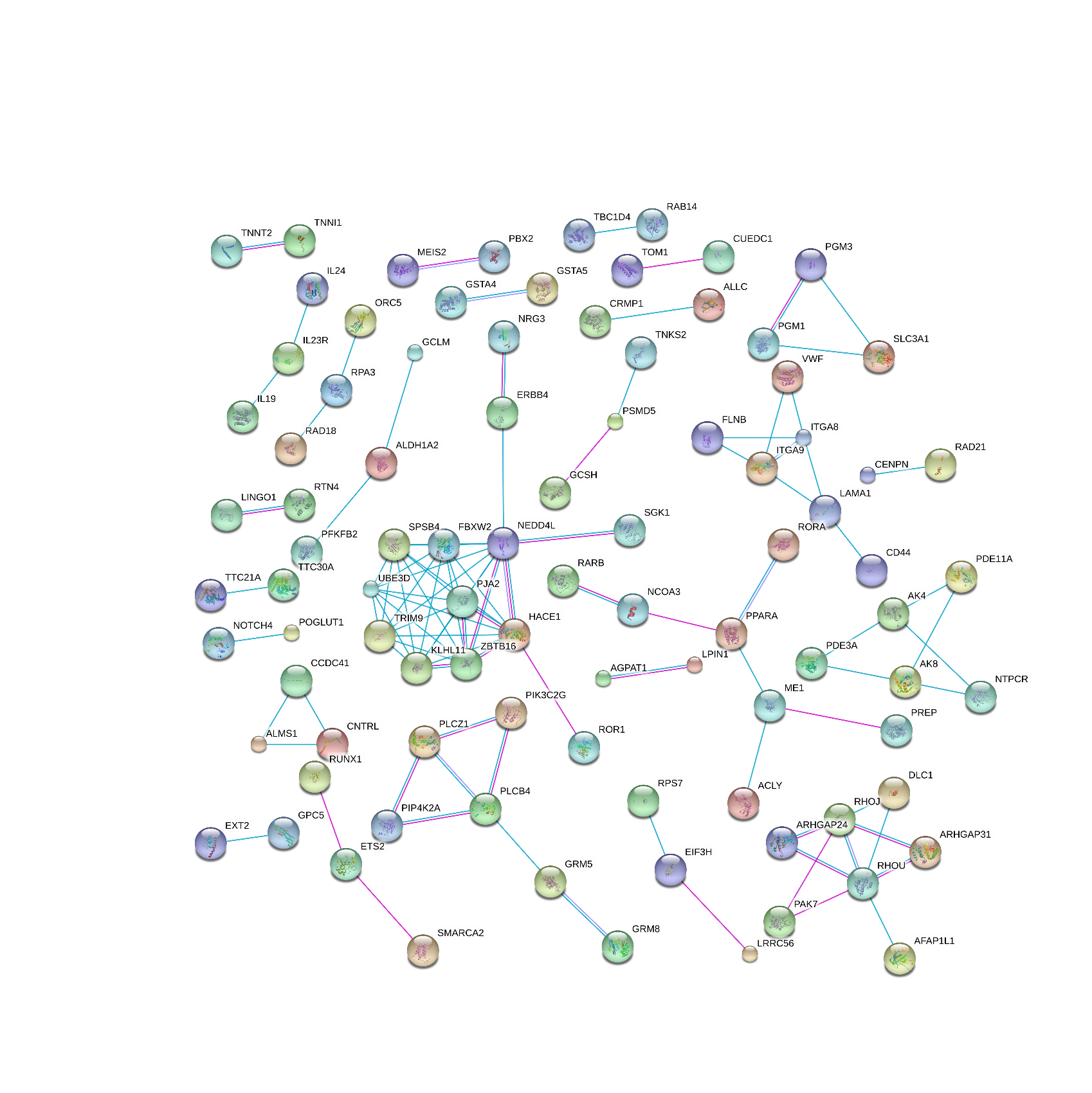
**

**Figure S7** Functionally related genes included the top 1% 200 kb windows ranked according to their proportion of outlier SNPs showing unusual nSL scores in the Central South Indian cluster. Only loci related by known interactions annotated in the STRING protein-protein interactions database are considered. Interactions determined experimentally are displayed by pink edges, while those inferred from literature information are displayed by light blue edges.

**
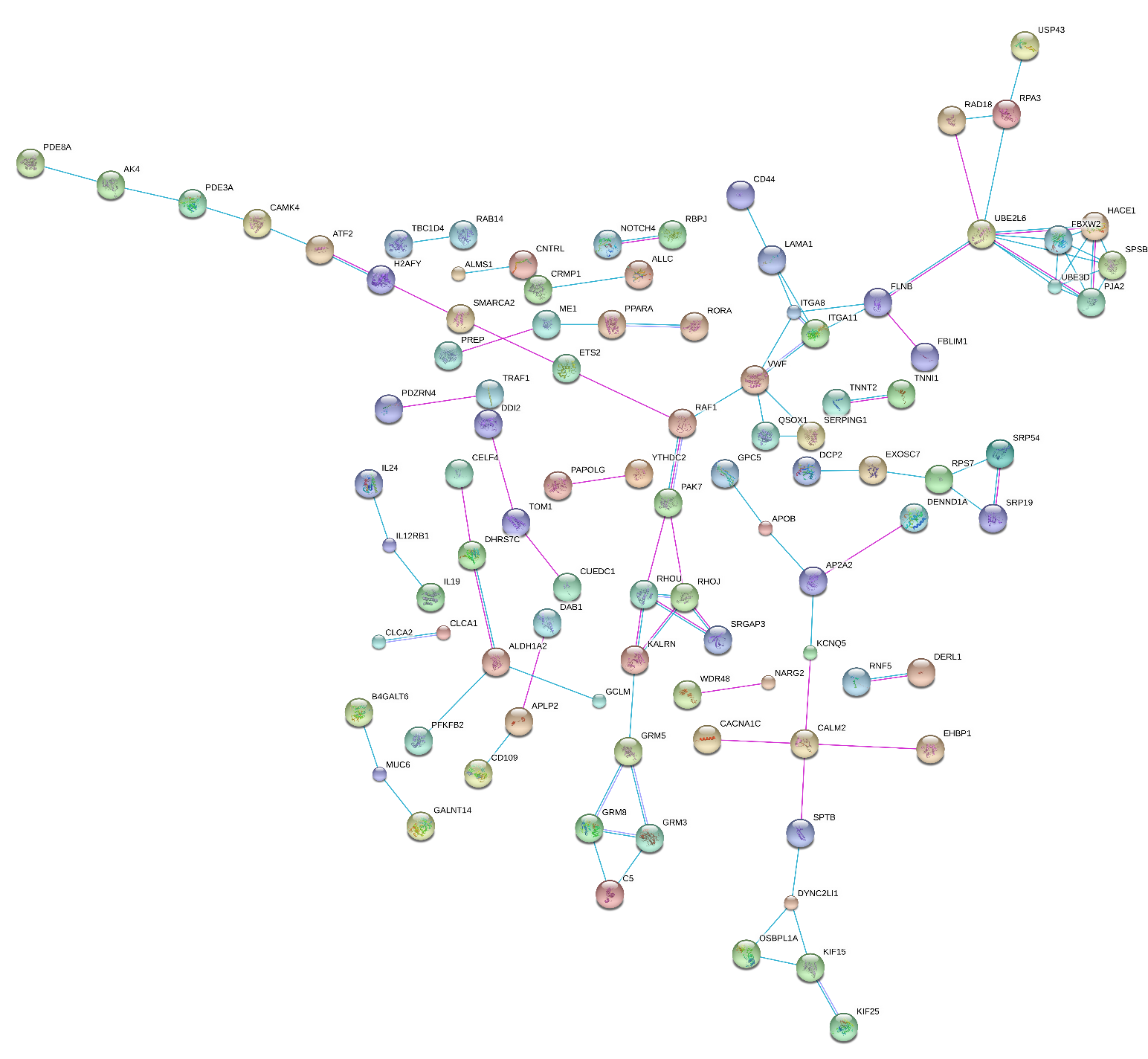
**

**Figure S8** Functionally related genes included the top 1% 200 kb windows ranked according to their proportion of outlier SNPs showing unusual nSL scores in the Bangladeshi cluster. Only loci related by known interactions annotated in the STRING protein-protein interactions database are considered. Interactions determined experimentally are displayed by pink edges, while those inferred from literature information are displayed by light blue edges.

**
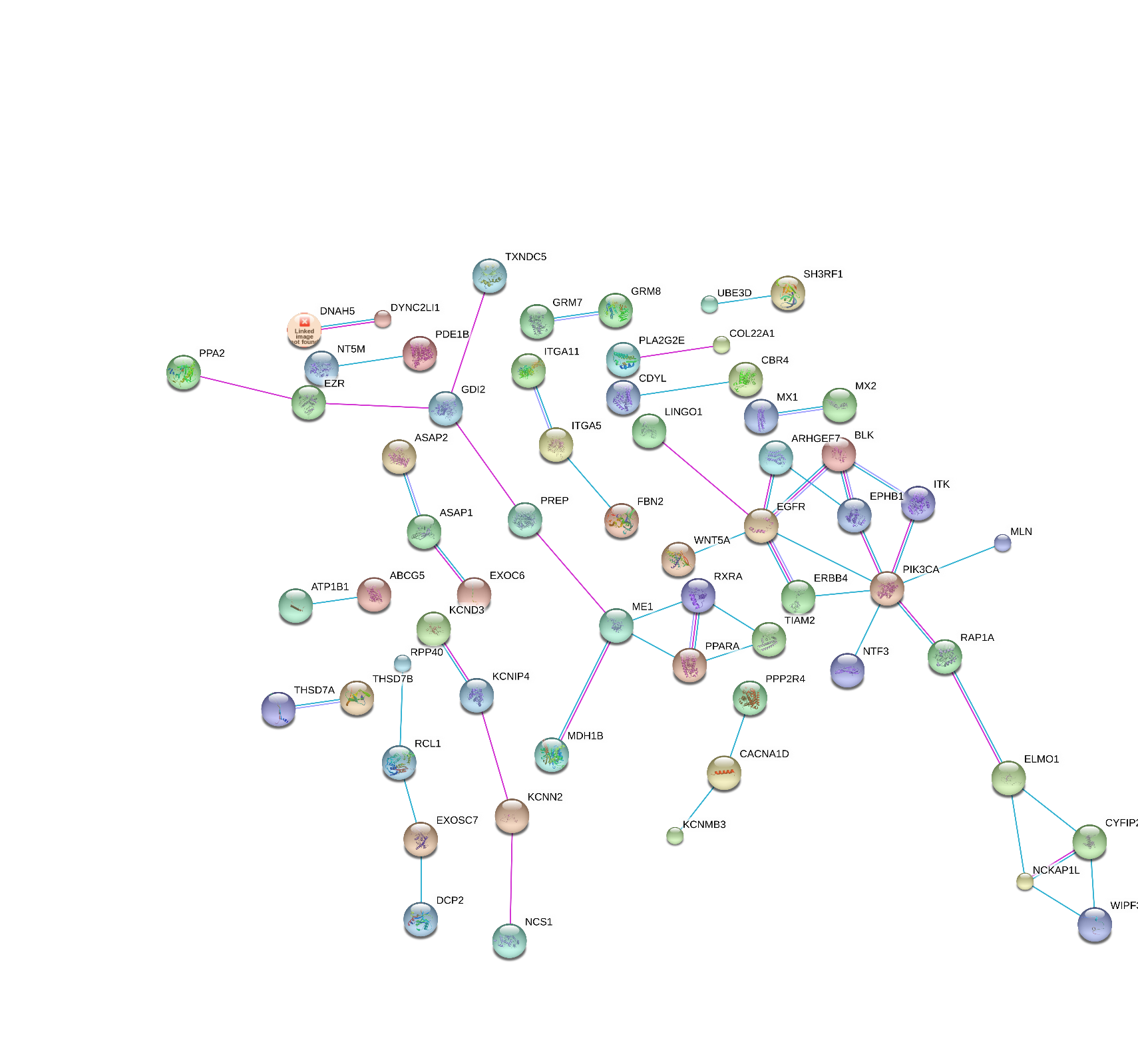
**

**Figure S9** Functionally related genes included the top 1% 200 kb windows ranked according to their proportion of outlier SNPs showing unusual nSL scores in the Tibeto-Buman cluster. Only loci related by known interactions annotated in the STRING protein-protein interactions database are considered. Interactions determined experimentally are displayed by pink edges, while those inferred from literature information are displayed by light blue edges.

**
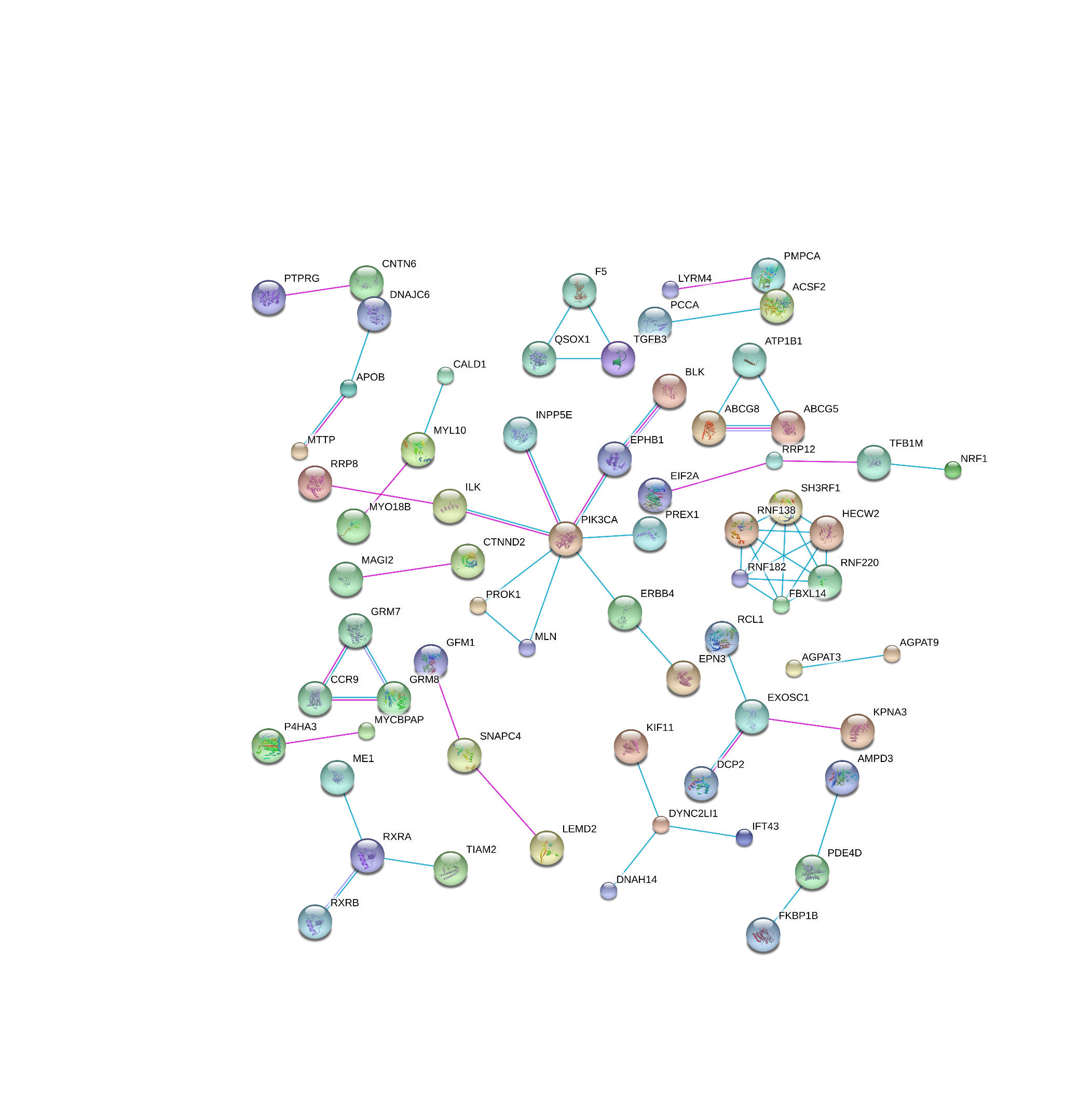
**

**Figure S10** Functionally related genes included the top 1% 200 kb windows ranked according to their proportion of outlier SNPs showing unusual nSL scores in the South East Asian cluster. Only loci related by known interactions annotated in the STRING protein-protein interactions database are considered. Interactions determined experimentally are displayed by pink edges, while those inferred from literature information are displayed by light blue edges.

**
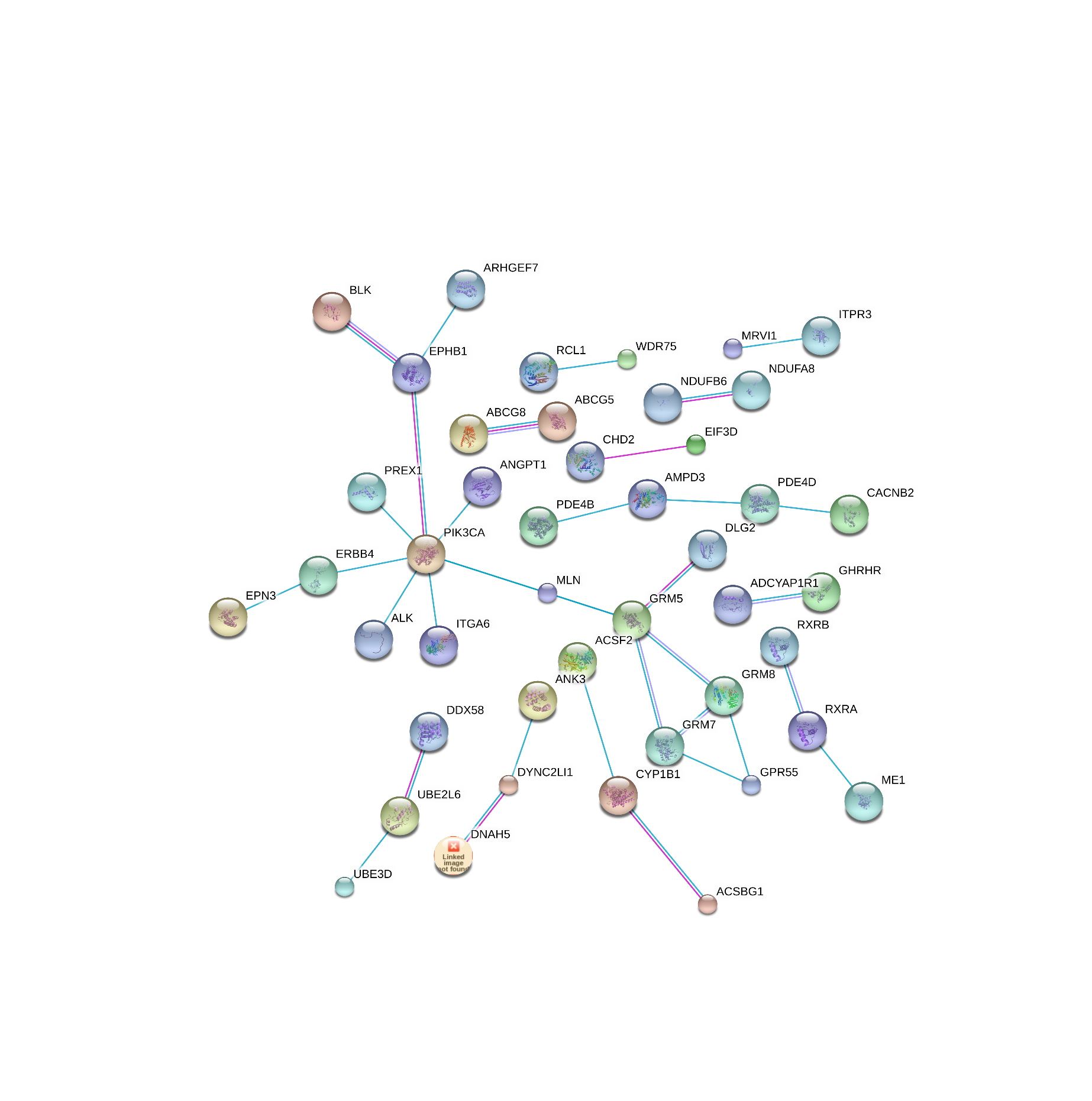
**

**Figure S11** Functionally related genes included the top 1% 200 kb windows ranked according to their proportion of outlier SNPs showing unusual nSL scores in the Dai-Vietnamese cluster. Only loci related by known interactions annotated in the STRING protein-protein interactions database are considered. Interactions determined experimentally are displayed by pink edges, while those inferred from literature information are displayed by light blue edges.


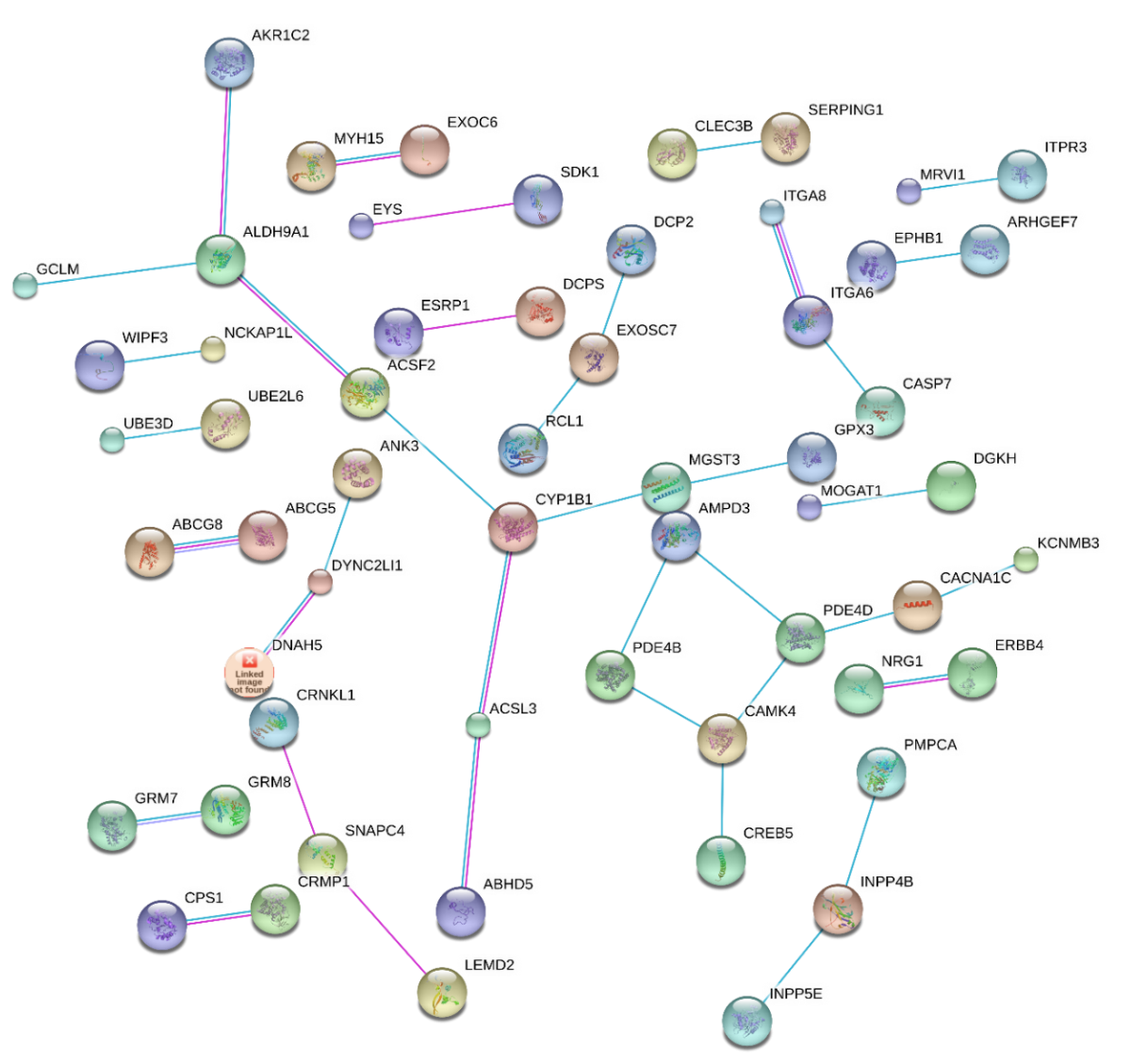


**Figure S12** Functionally related genes included the top 1% 200 kb windows ranked according to their proportion of outlier SNPs showing unusual nSL scores in the Han-Tujia cluster. Only loci related by known interactions annotated in the STRING protein-protein interactions database are considered. Interactions determined experimentally are displayed by pink edges, while those inferred from literature information are displayed by light blue edges.

**
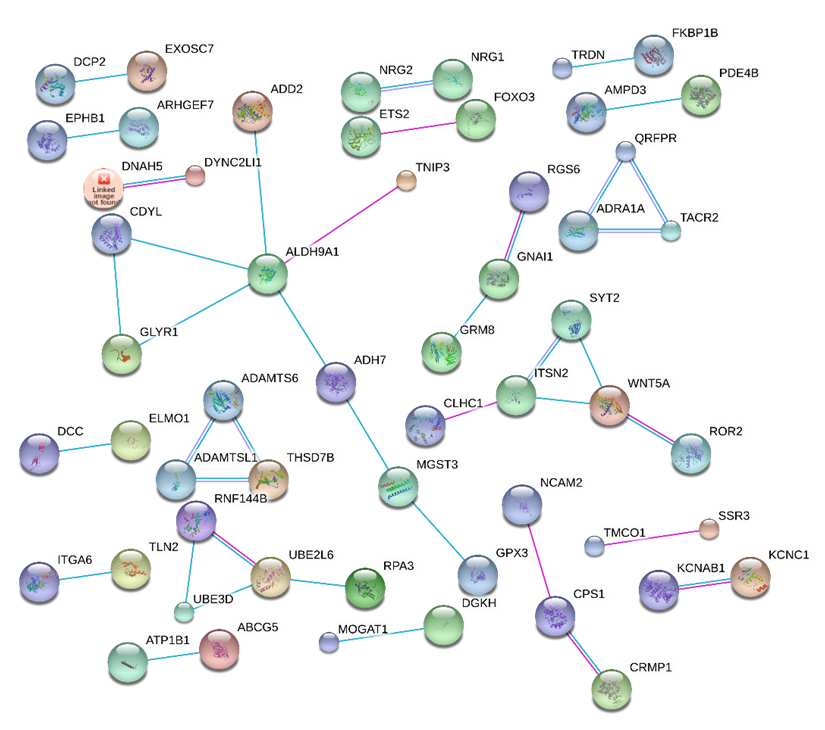
**

**Figure S13** Functionally related genes included the top 1% 200 kb windows ranked according to their proportion of outlier SNPs showing unusual nSL scores in the Japanese cluster. Only loci related by known interactions annotated in the STRING protein-protein interactions database are considered. Interactions determined experimentally are displayed by pink edges, while those inferred from literature information are displayed by light blue edges.


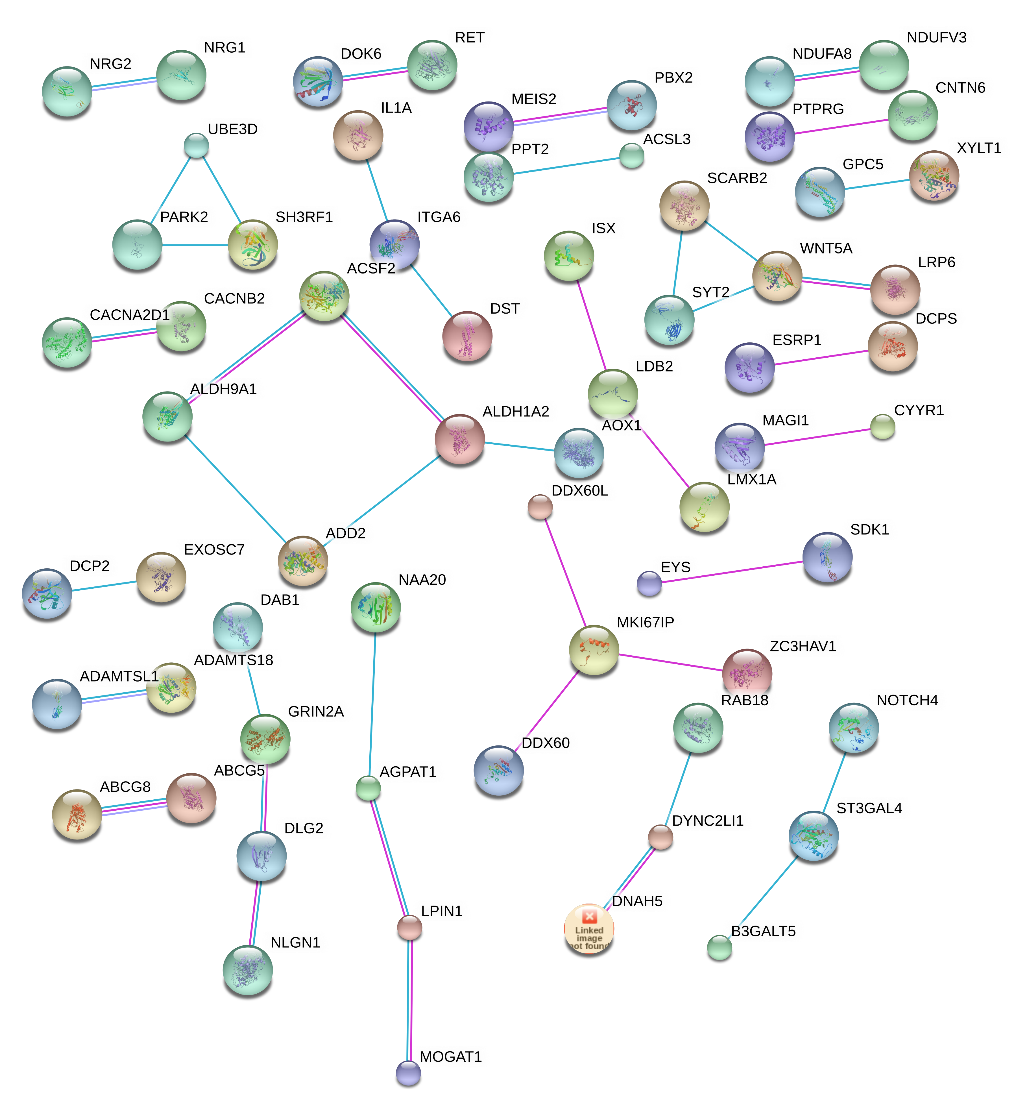


**Figure S14** Functionally related genes included the top 1% 200 kb windows ranked according to their proportion of outlier SNPs showing unusual nSL scores in the Korean cluster. Only loci related by known interactions annotated in the STRING protein-protein interactions database are considered. Interactions determined experimentally are displayed by pink edges, while those inferred from literature information are displayed by light blue edges.

**Table S3** Gene networks and related functional pathways showing evidence of pervasive positive selection in the CHB population.

| PATHWAY | PATHWAY SIZE | NETWORK SIZE | HSS | p-value | GENES |
| --- | --- | --- | --- | --- | --- |
| Glycerolipid metabolism | 51 | 13 | 6.302 | 0.013 | *DGKB, DGKG, DGKE, PLPP1, MGLL, LPIN1, AGPAT5, AGPAT3, GPAM, GPAT3, MOGAT1, PNLIPRP3, DGKH* |
| AGE-RAGE signaling in diabetic complications | 86 | 21 | 5.464 | 0.044 | *MAPK14, DIAPH1, KRAS, SMAD3, PLCB2, PLCB3, PLCB4, PRKCA, PRKCB, PRKCD, PRKCE, PRKCZ, MAPK1, MAPK3, MAPK10, MAPK13, RAC1, PLCB1, NOX3, PLCE1, PLCD4* |
| FoxO signaling | 114 | 17 | 5.953 | 0.022 | *CAT, CCND2, CCNG2, MAPK14, FOXO3, IL7R, MAPK1, MAPK3, MAPK10, MAP2K2, RAG1, CCNB2, BCL2L11, KLF2, PLK4, PLK2, GABARAPL1* |

**Table S4** Gene networks and related functional pathways showing evidence of pervasive positive selection in the JPT population.

| PATHWAY | PATHWAY SIZE | NETWORK SIZE | HSS | p-value | GENES |
| --- | --- | --- | --- | --- | --- |
| FoxO signaling | 114 | 21 | 5.983 | 0.021 | *BNIP3, CCND2, CCNG2, CDKN1A, MAPK14, S1PR1, FOXO1, FOXO3, IL7R, MAPK3, MAPK10, RAG1, SGK1, STAT3, TNFSF10, CCNB2, BCL2L11, KLF2, PLK4, PLK2, GABARAPL1* |
| Glycolysis/Gluconeogenesis | 58 | 9 | 5.679 | 0.032 | *ADH1A, ADH1B, ADH1C, ADH6, ADH7, ALDH1B1, ALDH3B1, ALDH9A1, ALDH7A1* |
| Glucagon signaling | 76 | 8 | 5.532 | 0.038 | *CREB1, ATF2, PRKACG, CREB5, PPARGC1A, CREB3L2, CREB3L1, CPT1C* |

**Supplementary Results**

**Inference of ancestry proportions for each individual genome**

ADMIXTURE analyses were performed on a subset of the “Pan-Asian” dataset made up of 1,171 samples belonging to 57 populations representative of the gradients of South Asian and East Asian variation pointed out by PCA and by testing K = 2 to K = 10 potential ancestral populations (Figure S2).

At K = 2, a clear differentiation between populations from the Indian subcontinent and other Asian groups was already observable due to the identification of two geographically restricted ancestry components. In fact, the vast majority of the examined populations showed a unique affiliation to one or the other genetic cluster, with the sole exception of Tibeto-Burman groups, which presented a predominant East Asian ancestry coupled with a reduced Indian component, as well as people from Bangladesh that showed the opposite admixture pattern (Figure S2). Instead, at K = 3 a further ancestry component appeared in the genetic background of Indian groups, reflecting the cline already pointed out by PCA. Indian populations indeed turned out to be characterized by a north-to-south gradient of ancestry components. In particular, Pakistani people (e.g. Balochi, Brahui and Makrani) were almost entirely characterized by the North Indian ancestry component differentially distributed from North to South India. Furthermore, in concordance with the geographic spread of the respective language groups, the Indian Indo-European and Dravidic speaking populations were also placed along a north to south cline (Metspalu et al., 2011), with the latter ones showing mainly a South Indian ancestry component, while the North Indian ancestry was significantly more represented in Indo-European groups (Figure S2). However, a recent study by Basu et al. (2015) has shown that the genomic structure of mainland Indian populations is best explained by contributions from four ancestral components. In addition to the previously mentioned Ancestral North Indian and Ancestral South Indian ones, proposed by Reich and colleagues (Reich et al., 2009), two other ancestral components that are predominant in the Tibeto-Burman speakers and Austro-Asiatic speaking tribes (denoted as Ancestral Tibeto-Burman and Ancestral Austro-Asiatic components), were identified respectively at K = 6 and K = 8 (Figure S2).

According to the performed ADMIXTURE analyses, a minor South Asian component was present not only in the mainland, but also in island Southeast Asian populations. Although this component was more widespread at lower Ks, at K = 8 the evidence was stronger for the Tibeto-Burman groups, such as the Burmese and Lahu, and slightly present also for the Malays. With the exception of the Burmese, who are geographically very close to the Indian subcontinent, this ancestry pointed to a rather reduced Indian gene flow, in contrast to the documented cultural influence. In fact, linguistic and archaeological evidence attest a continuous presence of South Asian cultures in Southeast Asia since 2,500 years ago, but probably driven by small groups of influential individuals from South Asia (Mörseburg et al., 2016).

When considering East and Southeast Asian populations, they displayed two main different ancestry components and such a distinction became visible at K = 4. The red ancestral component was found to be related to northern populations, being for instance the unique component represented in Japanese people and reaching high percentages also in Koreans, Mongolians, Oroqens, Hezhens and Daurs. On the other hand, the yellow ancestral component appeared to be related to southern populations, being in fact overwhelming among Cambodian, Dai, Lahu, Vietnamese and Austronesian groups (Figure S2). While in many populations the observed genomic ancestry was derived predominantly from one of the inferred components, in other groups multiple sources of ancestry could be inferred, as already reported by Li et al. (2008).

Previous studies have revealed strong genetic correlations between mainland and island South-East Asian populations that are geographically and linguistically close, suggesting a common origin of all South-East Asian and East Asian populations from a single migration wave (The HUGO Pan-Asian SNP Consortium, 2009). This pattern is corroborated in the present study by ADMIXTURE analysis testing K = 4 (Figure S2). However, it is well known that in the more recent past populations living in this region have undergone major demographic changes, particularly during the last 5,000 years in association with the Austronesian expansion (Mörseburg et al., 2016). In particular, this process, which related to the spread of the Neolithic culture and the Austronesian languages, represents a complex demographic puzzle characterized by the interaction between migrating Neolithic farmers and indigenous Mesolithic hunter-gatherer communities (Xu et al., 2012). The group most representative of the ancestry component involved in such population movements are the Igorots, who at K = 5 showed no signature of admixture at all (Figure S2). Other groups characterized significantly by this ancestral component are the Dusuns and Muruts, inhabiting the Borneo, as well as the Malays and some Filipino people (here specify as Luz and Vizaya).

At K = 6, we then observed a new ancestral component proper of the Sherpa people, which distinguished East Asian populations into two further population groups other than the previously described northern and southern ones (Wang et al., 2011; Jeong et al., 2014; Gnecchi-Ruscone et al., 2017). In particular, the Chinese Han and Tujia populations and the Tibeto-Burman groups exhibited this ancestry component, whereas Japanese, Dai, Vietnamese, Cambodians and Malaysian groups did not show such a Sherpa-like genetic contribution (Figure S2).

The last ancestral component that appeared at K = 8, the most plausible scenario, was almost fixed in the Birhor group, being also widespread, even if at low percentages, in all South Asian and in many South-East Asian populations (Figure S2).
